# Mechanistic study of the rhodium-catalyzed carboxylation of simple aromatic compounds with carbon dioxide†
†Electronic supplementary information (ESI) available: Experimental details and crystallographic data in cif format. CCDC 1017905. For ESI and crystallographic data in CIF or other electronic formats see DOI: 10.1039/c6sc03838g



**DOI:** 10.1039/c6sc03838g

**Published:** 2016-11-01

**Authors:** Takuya Suga, Takanobu Saitou, Jun Takaya, Nobuharu Iwasawa

**Affiliations:** a Department of Chemistry , Tokyo Institute of Technology , O-okayama, Meguro-ku , Tokyo 152-8551 , Japan . Email: niwasawa@chem.titech.ac.jp

## Abstract

A detailed mechanism of the Rh(i)-catalyzed carboxylation of simple aromatic compounds *via* C–H bond activation was investigated. Kinetic studies with model compounds of the postulated key intermediates revealed that 14-electron complexes, RhMe(dcype) and RhPh(dcype), participated in the C–H bond activation step and the carboxylation step, respectively. Interestingly, the undesired carboxylation of RhMe(dcype) to give acetic acid was found to be much faster than the desired C–H bond activation reaction under stoichiometric conditions, however, the C–H bond activation reaction could occur under catalytic conditions. Careful controlled experiments revealed that C–H bond activation using RhMe(dcype) became competitive with its direct carboxylation under the condition that the concentration of CO_2_ in the liquid phase was rather low. This factor could be controlled to some extent by mechanical factors such as the stirring rate and the shape of the reaction vessel. The resting state of the rhodium species under catalytic conditions was found to be [RhCl(dcype)]_2_, and the proposed intermediates such as RhMe(dcype) and Rh(OBz)(dcype) were readily converted to the most stable state, [RhCl(dcype)]_2_, *via* transmetallation with [Al]–Cl species, thus preventing the decomposition of the active catalytic species.

## Abstract 2

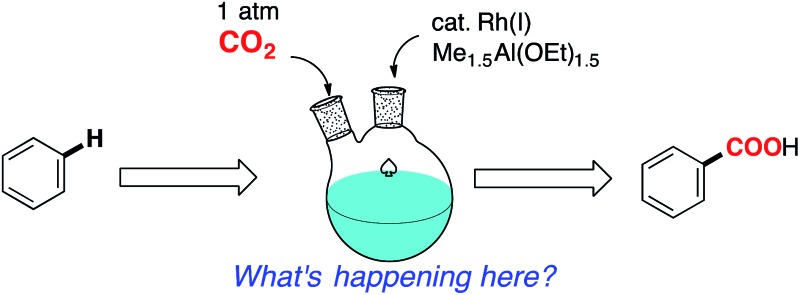
A detailed mechanistic study of the rhodium-catalyzed carboxylation of aromatic compounds was carried out to clarify the unique characteristics of this reaction.

## Introduction

**Scheme 1:**
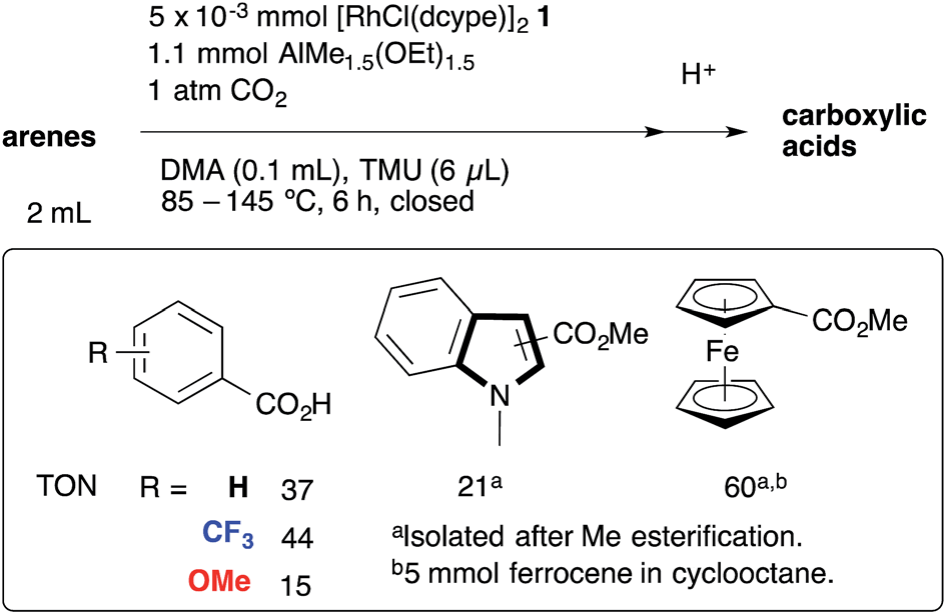
Rh-Catalyzed C–H bond carboxylation.

Catalytic direct carboxylation of simple hydrocarbons with carbon dioxide (CO_2_) is a formidable challenge in organic chemistry.^1^ Recently, we reported Rh(i)-catalyzed carboxylation of simple aromatic compounds such as benzene and toluene using a combination of [RhCl(dcype)]_2_ **1** (dcype: 1,2-bis(dicyclohexylphosphino)ethane) and AlMe_1.5_(OEt)_1.5_ as a stoichiometric methylating agent (Scheme 1).^2–4^ The most attractive feature of this reaction is its wide generality. Not only electron poor/rich arenes, but also heteroaromatics such as benzofuran and indole were carboxylated successfully. Furthermore, ferrocene showed remarkable reactivity in this reaction.

**Scheme 2:**
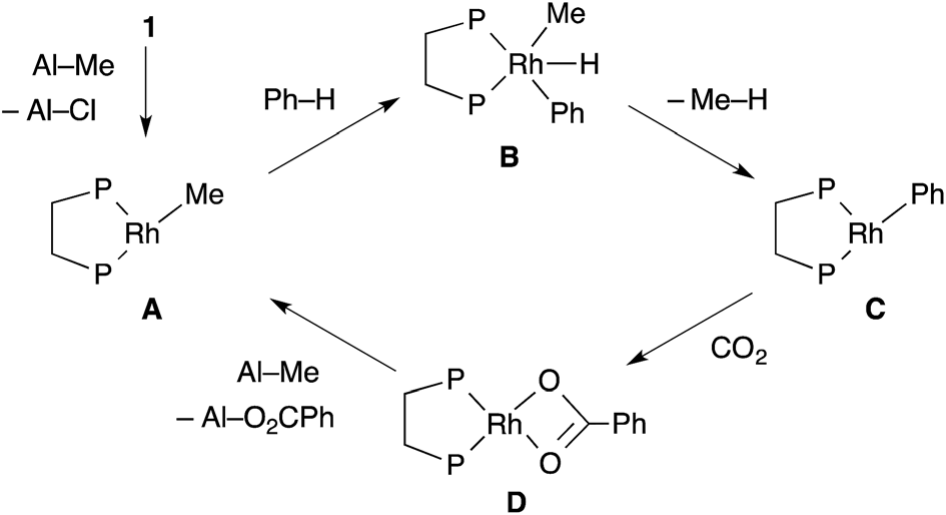
Postulated reaction mechanism.

The proposed reaction mechanism is shown in Scheme 2. The reaction starts with the generation of a 14-electron methylrhodium(i) complex **A** through transmetallation of [RhCl(dcype)]_2_ **1** with AlMe_1.5_(OEt)_1.5_, followed by oxidative addition of an sp^2^ C–H bond of benzene to **A**, giving phenyl(hydrido)(methyl)rhodium(iii) intermediate **B**. Reductive elimination of methane from **B** affords a reactive 14-electron phenylrhodium(i) complex **C**.^5^ Nucleophilic addition of **C** to CO_2_ gives a rhodium(i) benzoate complex **D**,^6^ which is converted to methylrhodium(i) **A** through transmetallation with AlMe_1.5_(OEt)_1.5_.

The key to our success consists of three main factors. The first is the choice of methylrhodium(i) as the C–H activation species. It was reported as a stoichiometric reaction by Andersen's and Field's groups that heating or photoirradiating methylrhodium(i) species in benzene afforded the corresponding phenylrhodium(i) species.^5^ It was expected that by using methylrhodium(i) species, the irreversible dissociation of methane from intermediate **B** would give the desired arylrhodium(i) species efficiently. The second is the reactivity of RhMe(dcype) **A**. For the success of the preparation of benzoic acid, direct carboxylation of **A** to produce acetic acid should be slower than the C–H bond activation of benzene by **A**. If the direct carboxylation was much faster than the C–H bond activation, the reaction would produce acetic acid only. The third one is the choice of the methylating agent to regenerate **A**. Methylaluminum was selected because it is widely known to be reactive for transmetallation, but it does not directly react with CO_2_ under appropriate conditions. Other reagents such as MeMgBr are unsuitable for this reason.

Apart from its synthetic utility, a noteworthy feature of this reaction is the realization of C–H bond activation followed by nucleophilic addition using the combination of a low-valence transition metal catalyst and a stoichiometric alkylating agent. Such C–H bond functionalization strategies are quite limited and most of the reported C–H activation-nucleophilic addition reactions utilize high-valence transition metal complexes such as Rh(iii) for C–H bond activation.^7^ In 2010, we reported the first example of such reaction, that is, rhodium-catalyzed direct carboxylation of 2-phenylpyridines.^8^ In 2012, Yoshikai reported a catalytic C–H bond activation of 2-phenylpyridine derivatives using the combination of a cobalt catalyst and a stoichiometric organomagnesium reagent, followed by nucleophilic addition to *N*-arylimines.^9^ Very recently, Wang reported that the use of a manganese catalyst with a stoichiometric amount of dimethylzinc was effective for the coupling of 2-phenylpyridines with aldehydes and nitriles.^10,11^ This kind of catalytic nucleophilic addition is still limited, but would become a powerful methodology in C–H bond functionalization reactions.

As described above, several reactions have recently been reported for this type of C–H activation-nucleophilic addition reactions, however, there has been almost no detailed study on the mechanism of such reactions, probably because of the difficulty in capturing highly reactive alkyl or aryl transition metal intermediates. For example, confirmation of the intermediacy of the 14-electron complexes in the C–H activation and carboxylation steps is not necessarily easy in our reaction because of their instability. As already described, stoichiometric reactivity of relevant methylrhodium(i) species to C–H bond activation has been known for more than three decades, but its detailed mechanistic study has not been carried out.^5^ Concerning the carboxylation step, there are several reports for stoichiometric carboxylation reactions of arylrhodium(i) complexes,^6^ however, their mechanisms have been proposed only by theoretical studies.^12^ Furthermore, transmetallation behaviors and resting states are also left unclarified. In this paper, we report a detailed analysis of the mechanism of this rhodium-catalyzed C–H carboxylation reaction based on kinetic studies using several model compounds, the examination of various reaction conditions including the shape of the reaction vessels and the stirring rate, analysis of the reaction mixture, and some controlled experiments.

## Results and discussion

### 1. Preparation and reactivity of tetracoordinated 16-electron rhodium species

To support the proposed reaction mechanism shown in Scheme 2, we initially tried to prepare each intermediate in the proposed catalytic cycle. In particular, 14-electron complexes RhMe(dcype) **A** and RhPh(dcype) **C** were the most attractive because they were thought to be true intermediates in the most important C–H bond activation and carboxylation steps.

**Scheme 3:**
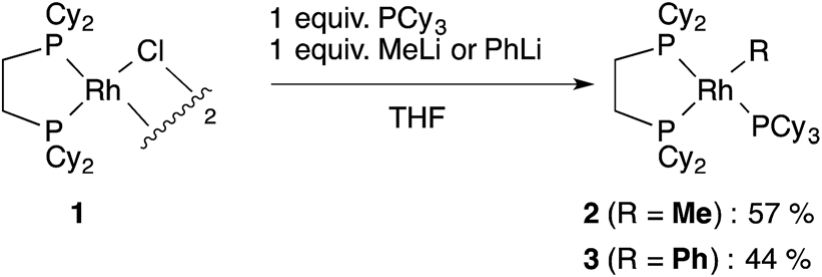
Preparation of model complexes.

Tricoordinated 14-electron alkylrhodium complexes are known to be so unstable that, up to now, they had not been isolated except for few specific examples.^13^ Indeed, we initially attempted to prepare them by treating [RhCl(dcype)]_2_ **1** with several methylating agents such as methyllithium, methylmagnesium bromide and trimethylaluminum, however, all of our efforts turned out to be fruitless. Therefore, we decided to prepare the tetracoordinated 16-electron complexes RhMe(PCy_3_)(dcype) **2** and RhPh(PCy_3_)(dcype) **3** as appropriate precursors of the 14-electron complexes (Scheme 3).^14^ **2** and **3** were prepared in good yields through the treatment of [RhCl(dcype)]_2_ with MeLi and PhLi, respectively in the presence of a stoichiometric amount of PCy_3_.^15^ Their structures were determined using ^1^H and ^31^P NMR. **3** was also characterized using single crystal X-ray structure analysis (see ESI†).

**Scheme 4:**
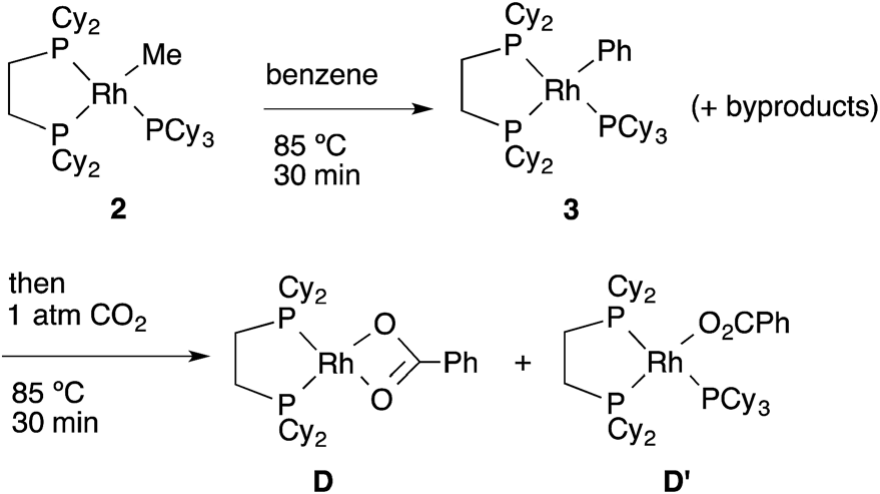
Reactions of model complexes.

Next, the reactivity of these complexes for the C–H bond activation and carboxylation reactions was examined. To our delight, RhMe(PCy_3_)(dcype) **2** was found to react with benzene to give RhPh(PCy_3_)(dcype) **3** under argon at 85 °C with perfect conversion although partial decomposition was also observed (Scheme 4). Moreover, further exposure of this solution to 1 atm CO_2_ at 85 °C gave a mixture of the carboxylated products Rh(O_2_CPh)(dcype) **D** and Rh(O_2_CPh)(PCy_3_)(dcype) **D′** (**D** : **D′** = 1 : 2 at room temperature). No intermediates were observed in these two reactions. The rhodium benzoates were characterized by comparing their ^31^P NMR spectra with those of authentic samples of **D** and **D′** (see ESI† for their preparative methods).

### 2. Mechanistic study on C–H bond activation by methylrhodium(i) species

**Fig. 1:**
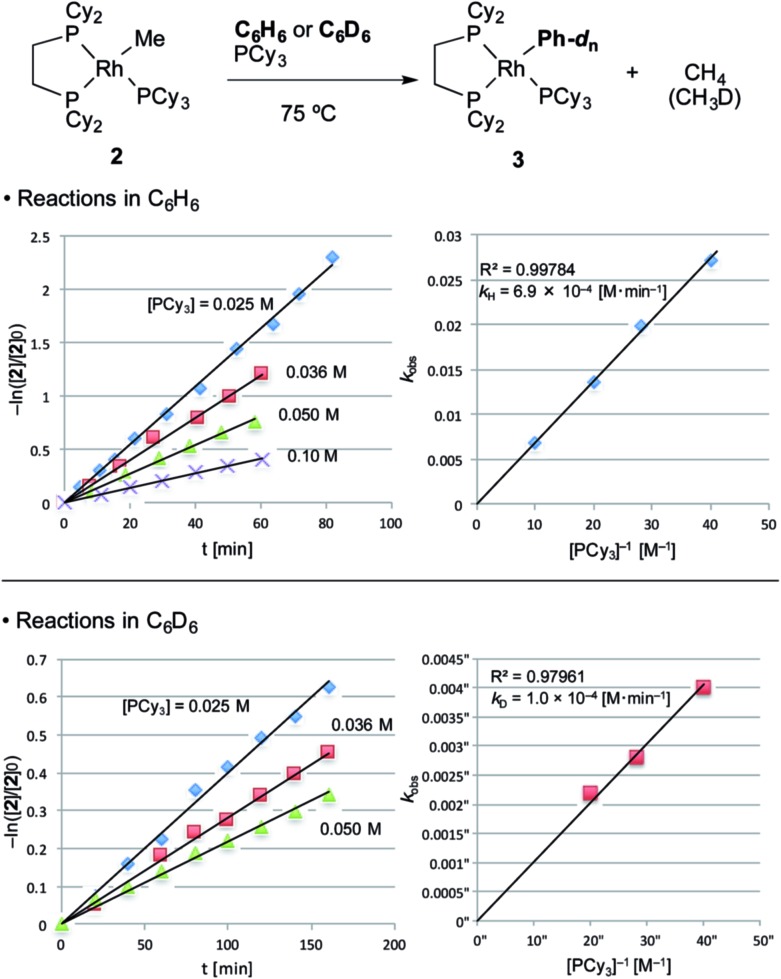
Kinetics of C–H bond activation. Conditions: 0.005 mmol **2**, 0.50 mL benzene in an NMR tube under argon. The reaction was analyzed using ^1^H NMR. Consumption of **2** was traced with the decay of the signal at *δ* = 0.4 (RhCH_3_). No other products were observed using ^31^P NMR after the reaction.

With the suitable model complexes in hand, we carried out a kinetic study of the C–H bond activation step by monitoring the reaction with ^1^H NMR (Fig. 1). The rate of the C–H bond activation reaction of RhMe(PCy_3_)(dcype) **2** with benzene-*d*_0_ as a solvent was measured in the presence of various concentrations of PCy_3_. Fortunately, the addition of PCy_3_ completely suppressed decomposition of the complex, and the desired transformation proceeded quantitatively according to the ^31^P NMR spectra (see ESI†).^16^ The reaction was found to be first order to RhMe(PCy_3_)(dcype) **2** and inverse first order to PCy_3_ (eqn (1)).

1 –ln([**2**]/[**2**]_0_) = *k*_H(D)_[PCy_3_]^–1^*t*

*k*_H_ = 6.9 × 10^–4^ [M min^–1^]*k*_D_ = 1.0 × 10^–4^ [M min^–1^]*k*_H_/*k*_D_ = 6.9

The value of –ln([**2**]/[**2**]_0_) was completely proportional to the reaction time even after 90% conversion, and was inversely proportional to [PCy_3_] with the rate constant *k*_H_ = 6.9 × 10^–4^ [M min^–1^] at 75 °C. According to the steady state approximation, this reaction includes dissociation of PCy_3_ before the rate-determining step. In addition, the reaction in benzene-*d*_6_ was apparently slower (*k*_D_ = 1.0 × 10^–4^ [M min^–1^]) than in benzene-*d*_0_ and the KIE value (*k*_H_/*k*_D_) was estimated to be 6.9. This large KIE value strongly suggests that the C–H bond activation step is rate-determining in this stoichiometric reaction.

**Scheme 5:**
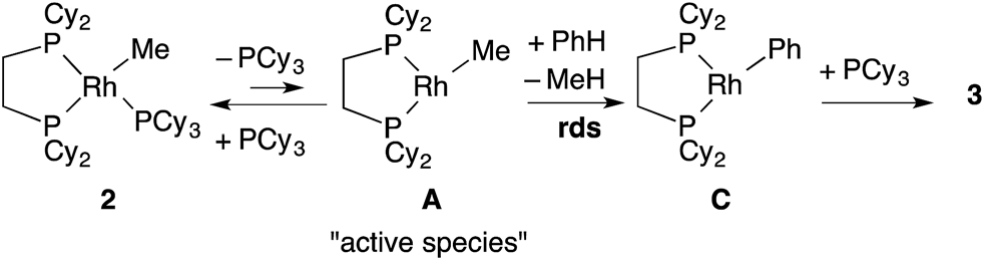
Plausible pathway of the C–H bond activation step.

With these results, it was concluded that tricoordinated RhMe(dcype) **A** is the true intermediate of the C–H bond activation step (Scheme 5). ^1^H NMR analysis also indicated the formation of CH_4_ in benzene-*d*_0_, and CH_3_D in benzene-*d*_6_. The detailed process of C–H bond activation remains unclear through our study, but the oxidative addition–reductive elimination process should be the most plausible. For instance, Sakakura reported that photo-induced reaction of Rh^I^Cl(PMe_3_)_3_ in benzene generated Rh^III^(H)(Cl)(Ph)(PMe_3_)_3_.^17,18^

**Fig. 2:**
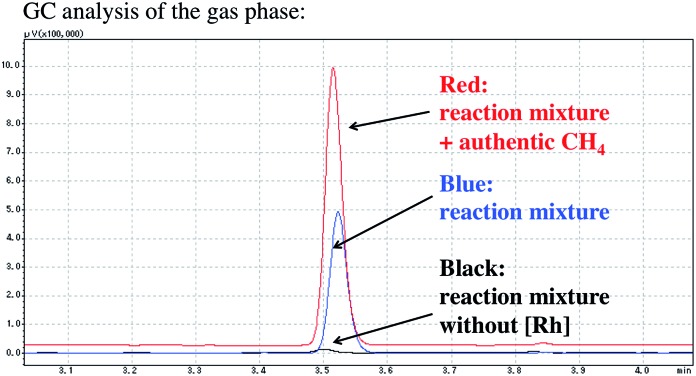
Methane observation using GC analysis. The reaction was carried out in benzene at 85 °C. Detailed reaction conditions were the same as shown in Scheme 1.

The formation of methane was also confirmed using GC analysis of the reaction mixture under catalytic conditions (Fig. 2). After the catalytic carboxylation of benzene was carried out under optimized conditions using [RhCl(dcype)]_2_ **1** and AlMe_1.5_(OEt)_1.5_, the gas phase of the resulting mixture was directly injected into the GC. While no methane formation was observed in the absence of [RhCl(dcype)]_2_ **1**, it was detected under catalytic conditions.

We also carried out several KIE studies under catalytic conditions to determine the turnover-limiting step. It should be noted that an accurate kinetic study of this reaction is difficult under our conditions because of the CO_2_ concentration problem (noted in the later section) and complex disproportionation of the methylaluminum reagent. Therefore, the results described below may not be very precise but are sufficient for general discussion.

**Scheme 6:**
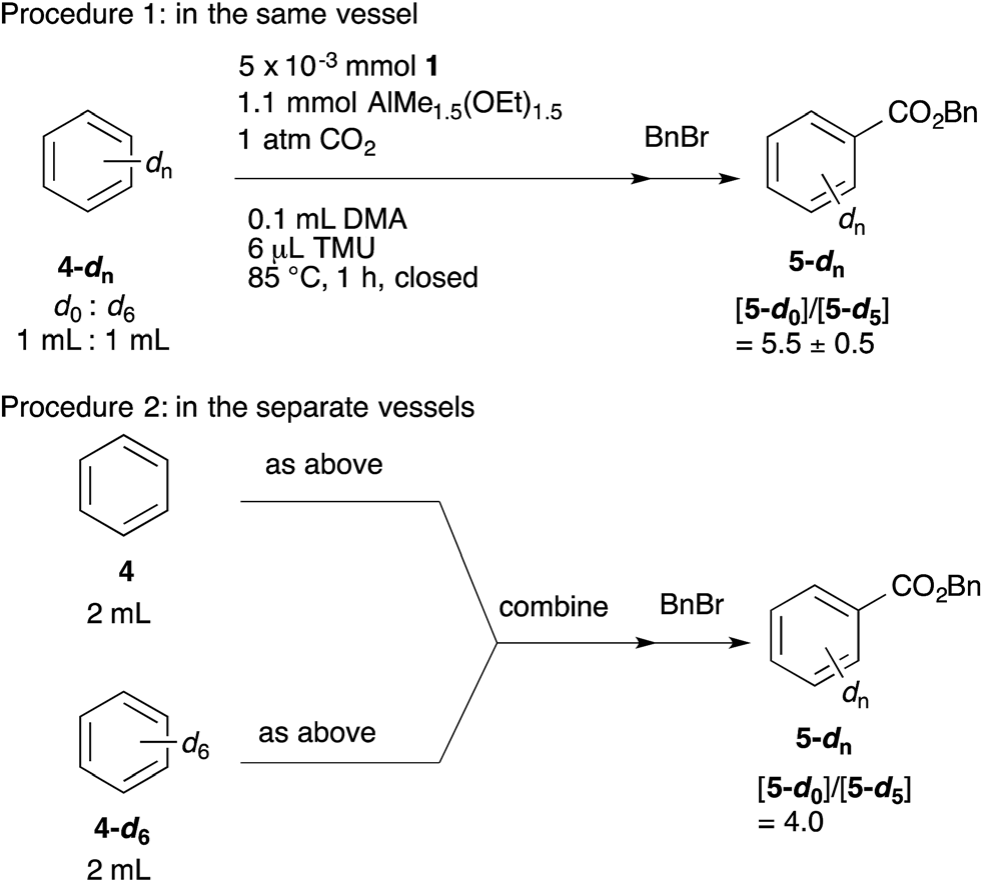
KIE study under catalytic conditions.

The KIEs were measured using two procedures (Scheme 6). In procedure 1, the reaction was performed using a 1 : 1 mixture of benzene-*d*_0_ and benzene-*d*_6_. In procedure 2, the reactions with benzene-*d*_0_ and benzene-*d*_6_ were carried out in separate vessels, and the resulting mixtures were combined after quenching the reaction with aqueous 1 M HCl. Both reactions were performed at 85 °C for 1 h. The ratio of benzoic acid-*d*_0_ and benzoic acid-*d*_5_ was estimated using ^1^H NMR after esterification of the benzoic acids with benzyl bromide. As a result, [**5-*d*_0_**]/[**5-*d*_5_**] = 5.5 (procedure 1) and [**5-*d*_0_**]/[**5-*d*_5_**] = 4.0 (procedure 2) were obtained. These large KIE values indicate that the C–H bond activation step is also the turnover-limiting step under catalytic conditions.

### 3. Mechanistic study of the carboxylation step

**Fig. 3:**
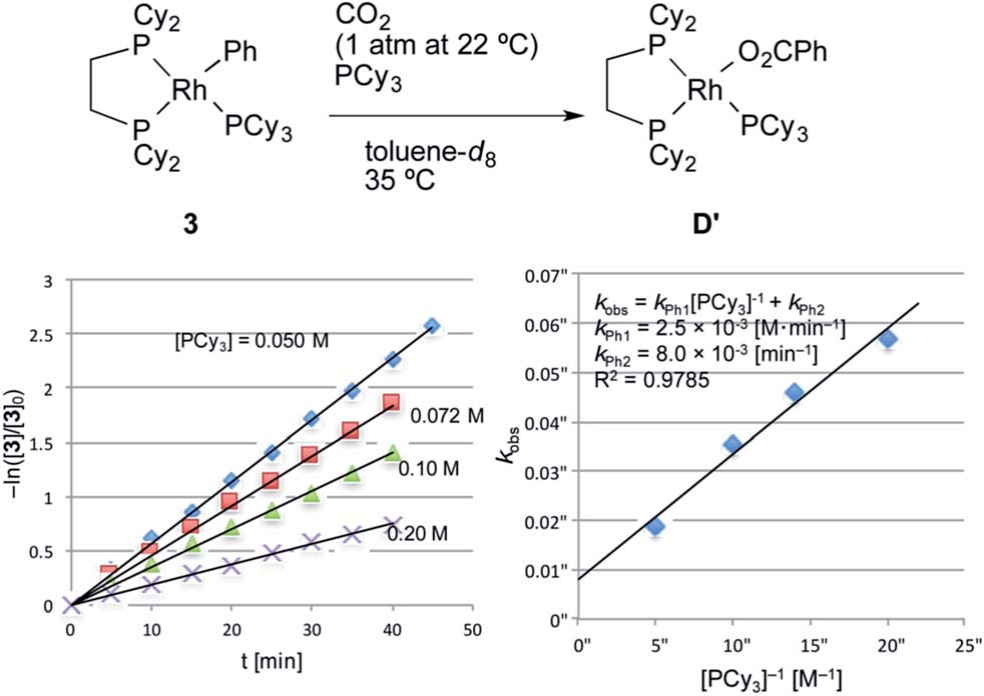
Kinetics of the carboxylation of **3**. Conditions: 0.005 mmol **3**, 0.50 mL toluene-*d*_8_ in an NMR tube (*ca.* 4 mL vol.) under 1 atm CO_2_. The solution was saturated with CO_2_ at 22–23 °C beforehand (see ESI†). Consumption of **3** was traced with the decay of the signal at *δ* 7.9 ppm (Ph). No other products were observed using ^31^P NMR after the reaction.

Next, a kinetic study of the carboxylation reaction of RhPh(PCy_3_)(dcype) **3** was carried out under CO_2_ (1 atm at 22–23 °C) in the presence of PCy_3_ in toluene-*d*_8_ (Fig. 3).^19^ The value of –ln([**3**]/[**3**]_0_) was proportional to the reaction time and was almost inversely proportional to [PCy_3_] with the rate constant *k*_Ph1_ = 2.5 × 10^–3^ [M min^–1^] at 35 °C (eqn (2)), suggesting that the carboxylation step is much faster than the C–H activation step, which coincided with the results of the KIE studies.

2 –ln([**3**]/[**3**]_0_) = (*k*_Ph1_[PCy_3_]^–1^ + *k*_Ph2_)*t*

*k*_Ph1_ = 2.5 × 10^–3^ [M min^–1^]*k*_Ph2_ = 8.0 × 10^–3^ [min^–1^]

**Scheme 7:**
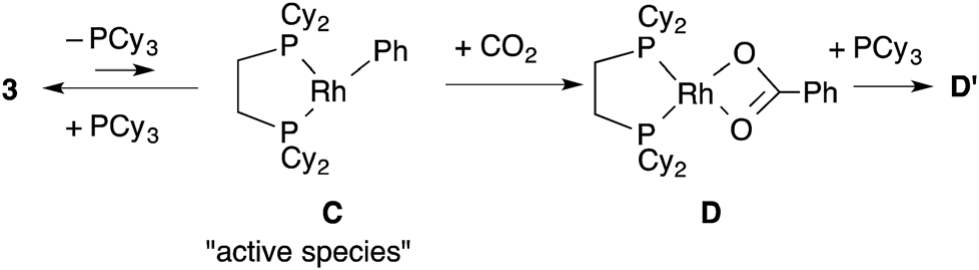
Plausible pathway of the carboxylation step.

Dissociation of PCy_3_ was also clearly involved in the carboxylation step. The small intercept term *k*_Ph2_ = 8.0 × 10^–3^ [min^–1^] left the possibility of a minor pathway (*e.g.* the reaction without dissociation of PCy_3_), but it was trivial at lower concentrations of PCy_3_. Therefore, it is concluded that the carboxylation step mainly proceeded *via* 14-electron complex RhPh(dcype) **C** in a similar manner to the C–H bond activation step (Scheme 7).

**Fig. 4:**
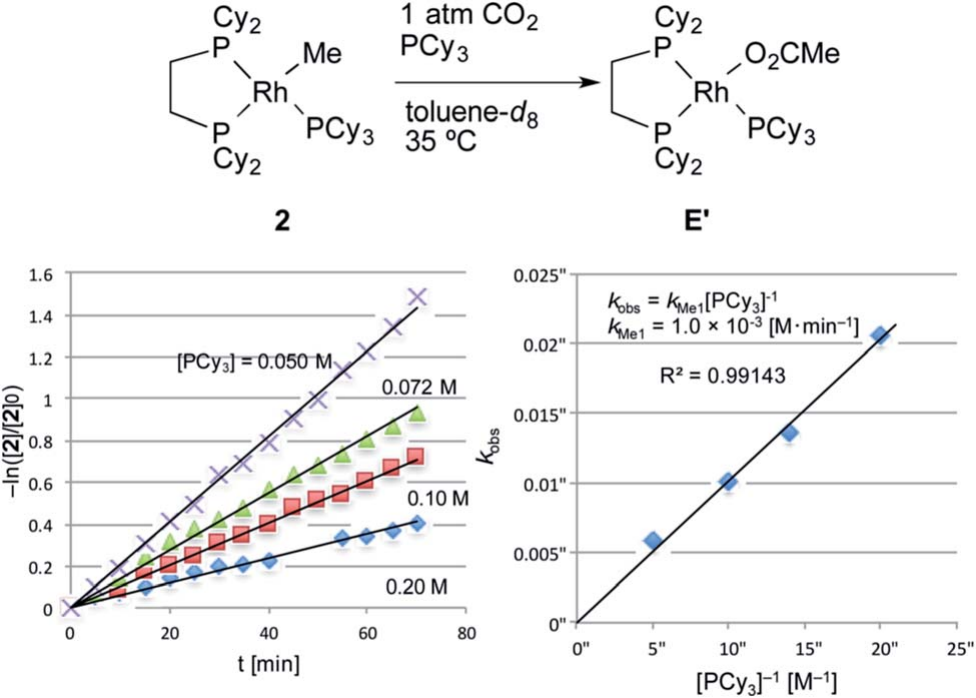
Kinetics of the carboxylation of **2**. Conditions: 0.005 mmol **2**, 0.50 mL toluene-*d*_8_ in an NMR tube (*ca.* 4 mL vol.) under 1 atm CO_2_. The solution was saturated with CO_2_ at 22–23 °C beforehand (see ESI†). Consumption of **2** was traced with the decay of the signal at *δ* 0.4 ppm (CH_3_).

The carboxylation reaction of RhMe(PCy_3_)(dcype) **2** was also carried out in toluene-*d*_8_ at 35 °C (Fig. 4).^20^ **2** showed a somewhat lower reactivity of carboxylation compared to RhPh(PCy_3_)(dcype) **3** (eqn (3), *k*_Me1_ = 1.0 × 10^–3^ [M min^–1^] *vs. k*_Ph1_ = 2.5 × 10^–3^ [M min^–1^]).

3 –ln([**2**]/[**2**]_0_) = *k*_Me1_[PCy_3_]^–1^*t*

*k*_Me1_ = 1.0 × 10^–3^ [M min^–1^]

**Scheme 8:**
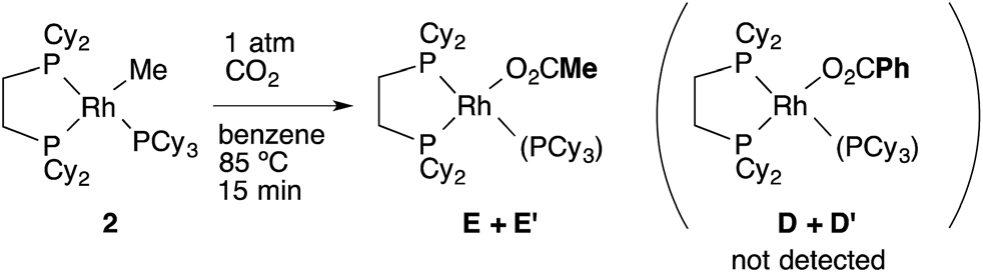
Stoichiometric carboxylation of **2** at 85 °C.

The equation was similar to that of RhPh(PCy_3_)(dcype) **3** except for complete disappearance of the intercept term (*k*_Me2_ = 0), suggesting the intermediacy of RhMe(dcype) **A** as a reactive species. ^1^H and ^31^P NMR indicated that there was no formation of the C–H activation product Rh(tol)(PCy_3_)(dcype) or corresponding benzoates throughout the reaction. In addition, the stoichiometric reaction of RhMe(PCy_3_)(dcype) **2** under 1 atm CO_2_ in benzene at 85 °C did not give benzoates at all, but gave only acetates Rh(OAc)(dcype) **E** and Rh(OAc)(PCy_3_)(dcype) **E′** (Scheme 8). These results imply that the carboxylation of RhMe(dcype) **A** with CO_2_ proceeded much faster than the C–H activation of **A** with benzene under 1 atm CO_2_. In other words, predominant formation of acetic acid should associate with the formation of benzoic acid.

**Scheme 9:**
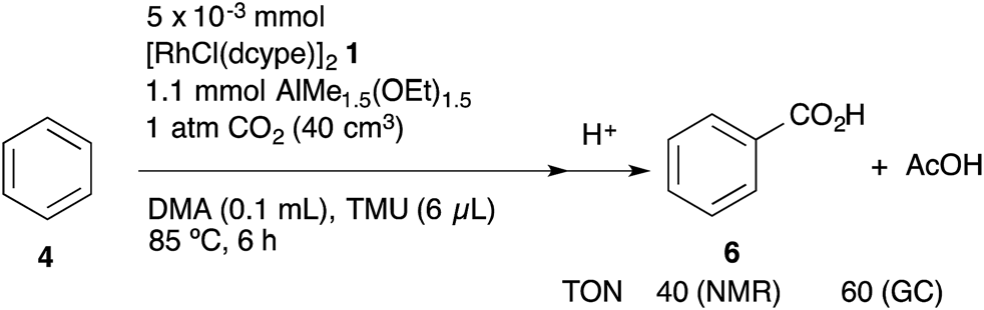
Confirmation of acetic acid formation.

Based on the above considerations, the TON of acetic acid (AcOH) was estimated using GC analysis under catalytic conditions. According to the standard catalytic procedure, the reaction was carried out in a 40 cm^3^ test tube for 6 h at 85 °C with the tube kept closed. As we expected, it was found that in addition to *ca.* 0.40 mmol (TON = 40) of benzoic acid, *ca.* 0.6 mmol (TON = *ca.* 60) of acetic acid was produced as judged using GC analysis (Scheme 9). However, the ratio of BzOH : AcOH = 2 : 3 was still inconsistent with the result of the stoichiometric study. To obtain more information on the competitive formation of benzoic acid and acetic acid, we decided to carry out a more detailed analysis of the reactions to clarify the difference of these stoichiometric and catalytic reactions.

All the reactions so far have been carried out using 40 cm^3^ test tube (*ca.* 2 mmol of CO_2_ should be inside) in a closed system for the catalytic reaction, and *ca.* 1 mmol of CO_2_ was consumed to produce benzoic acid and acetic acid as shown in Scheme 9. As the amount of CO_2_ in the reaction mixture was thought to be influential on the ratio of carboxylic acids under catalytic conditions, the reaction was examined using several vessels with different shapes and volumes.

The effect of the reaction vessel was examined by changing the total volume and/or the shape of the reaction vessel (Table 1).^21^ The reaction time was set to 1 h to reduce the effects of catalyst decomposition and reagent consumption. The stirring rate was kept at *ca.* 800 rpm. The reaction vessels were; a 40 cm^3^ test tube (*ø* = 2 cm, vessel 1) with a closed system (entry 1), a 40 cm^3^ test tube (vessel 1) with a 2000 cm^3^ balloon filled with CO_2_ to disregard the decrease of CO_2_ in a whole vessel (entry 2), a 40 cm^3^ round-bottom flask (vessel 2) with a 2000 cm^3^ balloon (entry 3) and a 160 cm^3^ round-bottom flask (vessel 3) with a closed system (entry 4). In entry 4, the content of CO_2_ should also be sufficient (*ca.* 7 mmol).

**Table 1:** Effect of the CO_2_ content and shape of the vessel^*a*^

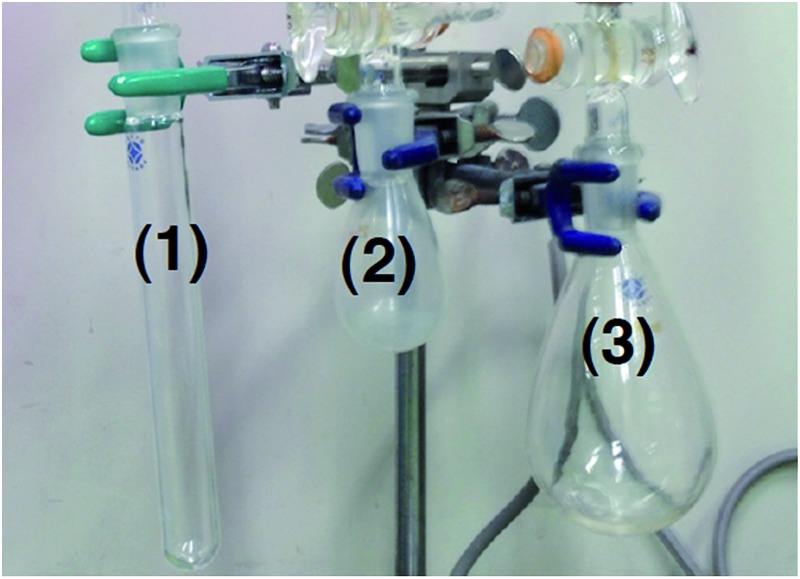					
Entry	Conditions	Total volume (cm^3^)	TON		[BzOH]/[AcOH]
			[BzOH]	[AcOH]	
1	Vessel 1	40	7.9	68	0.12
2	Vessel 1 + balloon	40 + 2000	5.7	42	0.14
3	Vessel 2 + balloon	40 + 2000	2.5	84	0.03
4	Vessel 3	160	1.3	147	0.01

^*a*^Vessel 1: 40 cm^3^ test tube (*ø* = 2 cm), vessel 2: 40 cm^3^ round-bottom flask, vessel 3: 160 cm^3^ round-bottom flask. Conditions are shown in Scheme 9, except the reaction time was shortened to 1 h. Stirring rates were approx. 800 rpm.

It was found that the volume of the vessel was not so influential on the ratio of benzoic acid and acetic acid, but the shape of the vessel caused a dramatic difference. The value of [BzOH]/[AcOH] in vessel 1 was 0.12 without a balloon and 0.14 with a balloon (entry 1 *vs.* 2). This indicated that the total content of CO_2_ was not responsible for the selectivity during the initial 1 h of reaction time.^22^ In sharp contrast, the use of vessel 2 decreased [BzOH]/[AcOH] to 0.03 (entry 2 *vs.* 3). The formation of acetic acid was predominant when vessel 3 was employed (entry 4, [BzOH]/[AcOH] = 0.01) as observed in the reaction of RhMe(PCy_3_)(dcype) **2** under CO_2_ in benzene (Scheme 8).

**Fig. 5:**
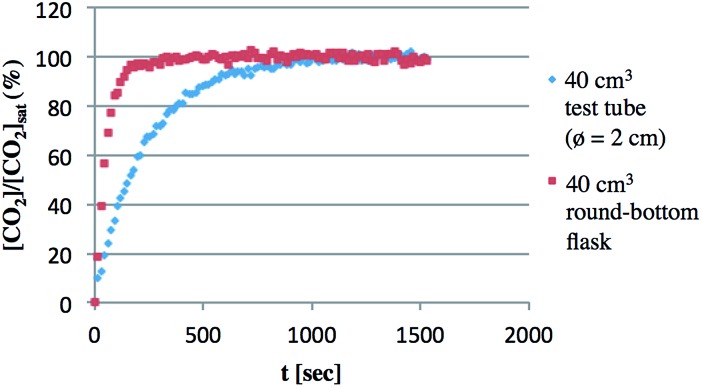
Dissolution of CO_2_ in toluene. Dissolution of CO_2_ was traced using *in situ*-IR spectrometry (2350 cm^–1^) in 2 cm^3^ toluene at room temperature. The mixtures were stood without stirring during measurement.

This large effect of the shape of the vessel would be due to the dissolution rate of CO_2_ into solution, which was elucidated using *in situ*-IR analysis at room temperature (Fig. 5).^23^ CO_2_ dissolution occurred more rapidly in a 40 cm^3^ round-bottom flask than in a 40 cm^3^ test tube even though they have almost the same total volume. For instance, the CO_2_ concentration reached saturation within 200 seconds in the round-bottom flask. On the other hand, it took almost 1000 seconds in the test tube. Although the results shown here were obtained without stirring, the rate of dissolution of CO_2_ certainly depends on the surface area of the solution, which influenced the ratio of BzOH and AcOH. For the same reason, the stirring rate was also found to be responsible (Table 2). Slower stirring gave an improved ratio of [BzOH]/[AcOH], although the total TON decreased. For example, [BzOH]/[AcOH] was 0.25 and the total TON was 41 at 100 rpm, while [BzOH]/[AcOH] was 0.07 and the total TON was 99 at 1000 rpm.

**Table 2:** Effect of stirring rate^*a*^

Entry	Rate [rpm]	TON		[BzOH]/[AcOH]
		[BzOH]	[AcOH]	
1	100	8.3	33	0.25
2	800	7.9	68	0.12
3	1000	6.2	93	0.07

^*a*^Reactions were carried out in 40 cm^3^ test tubes (closed system). Conditions were the same as noted in Table 1.

From these results, it was concluded that this reaction strongly depends on CO_2_ concentration in the liquid phase, which was mainly determined by the dissolution rate of CO_2_. In other words, the liquid phase is not saturated with CO_2_ owing to its fast consumption by carboxylation reactions under catalytic conditions. In the presence of sufficient CO_2_, intermediate RhMe(dcype) **A** mostly reacts with CO_2_ to give acetic acid before it reacts with benzene to give RhPh(dcype) **C**, as observed in entry 4 in Table 1 and entry 3 in Table 2, and this agrees with the result of the stoichiometric reaction of RhMe(PCy_3_)(dcype) **2** in CO_2_-saturated benzene. However, the true concentration of CO_2_ should be rather low under catalytic conditions particularly with slower stirring and a liquid phase with a smaller surface area. Carboxylation and C–H bond activation of benzene by RhMe(dcype) **A** becomes competitive under such conditions, and both benzoic acid and acetic acid were obtained catalytically in contrast to the stoichiometric reaction of RhMe(PCy_3_)(dcype) **2**.

In summary, we proved that 14-electron tricoordinated RhPh(dcype) **C** is a plausible intermediate in the carboxylation step. RhMe(dcype) **A** also reacted with CO_2_ in a similar way to give acetic acid catalytically. This undesired pathway was predominant over the desired C–H bond activation reaction under 1 atm CO_2_. Nevertheless, C–H bond activation of benzene took place successfully under catalytic conditions because the concentration of CO_2_ in the liquid phase was much lower than saturation due to its consumption by the carboxylation reaction of RhMe(dcype) **A**. Thus, the dissolution rate of CO_2_ controlled the fate of the key intermediate RhMe(dcype) **A**. This clearly indicates that the balance between C–H bond activation and the subsequent transformation is very important for the catalytic C–H bond functionalization reaction using these alkyl metal complexes.

### 4. Transmetallation behaviors

**Scheme 10:**
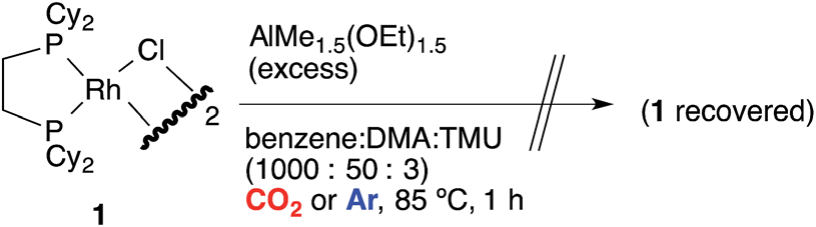
Analysis of the reaction mixture using ^31^P NMR.

Direct observation of the reaction mixture is a reliable method to obtain information on the resting state of the catalytic cycle. We then observed ^31^P NMR to clarify the rhodium species present in the reaction mixture after the catalytic reaction was carried out in benzene for 1 h under 1 atm CO_2_ at 85 °C (Scheme 10). It was a surprise to find that almost no complex except for the starting [RhCl(dcype)]_2_ **1** was observed even though benzoic acid was produced gradually. Alternatively, under argon only **1** was observed. No methylrhodium or phenylrhodium species were observed. [RhCl(dcype)]_2_ **1** mostly decomposed after 6 h of heating under Ar. Therefore, it was speculated that there would be equilibria between **A–D** and **1**, so that only **1** was observable.

**Scheme 11:**
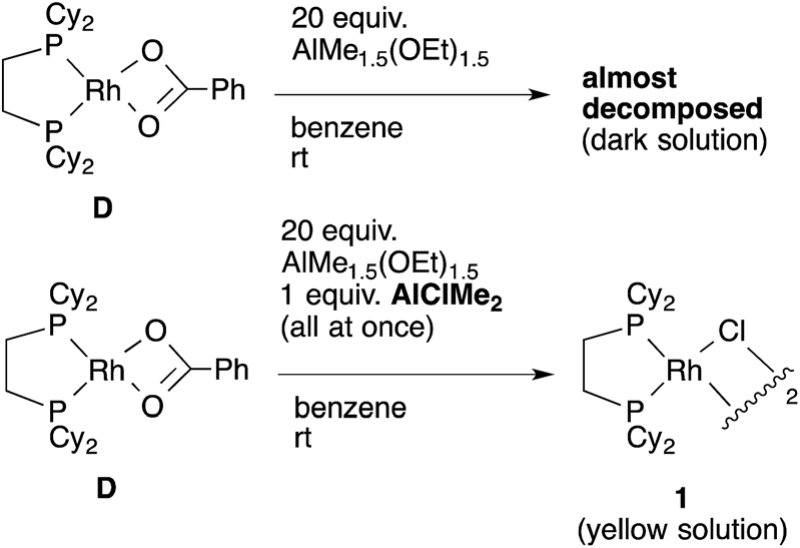
Transmetallation of Rh–O_2_CPh complex.

To confirm our speculation, Rh(O_2_CPh)(dcype) **D** was prepared and its catalytic activity was examined. When the catalytic carboxylation reaction of benzene was carried out using **D** as a catalyst, benzoic acid was obtained with a TON of only 2.5. In addition, when **D** and excess AlMe_1.5_(OEt)_1.5_ were mixed in benzene at room temperature (no chloride source), rapid decomposition of the complex was observed using ^31^P NMR (Scheme 11). However, it was found that addition of only an equimolar amount of AlClMe_2_ to **D** prevented it from decomposition to give [RhCl(dcype)]_2_ **1**, and the catalytic activity was recovered (TON = 28). In other words, the success of the present reaction was due to the fast transmetallation of rather unstable [Rh]–O_2_CPh or [Rh]–Me with [Al]–Cl to give a stable dimeric [Rh]–Cl species under catalytic conditions.

**Scheme 12:**
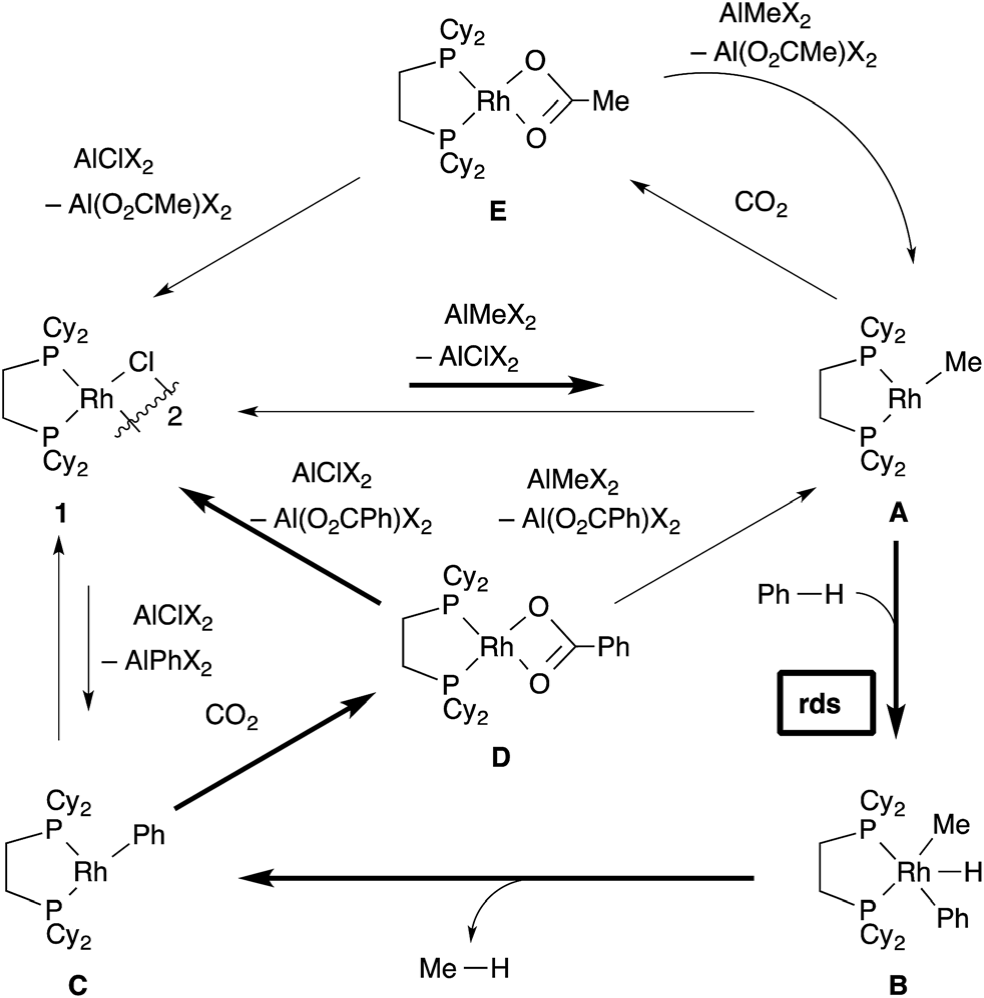
Total figure of the catalytic cycle.

The total figure of this reaction is illustrated in Scheme 12. The most important cycle, which provides benzoic acid, is confirmed to be fundamentally the same as we postulated (depicted in bold arrows). First, transmetallation of [RhCl(dcype)]_2_ **1** and AlMe_1.5_(OEt)_1.5_ generates the 14-electron methylrhodium complex, RhMe(dcype) **A**. **A** reacts with benzene to give the 14-electron phenylrhodium complex, RhPh(dcype) **C**, possibly *via* reductive elimination of methane from Rh(H)(Me)(Ph)(dcype) **B**. The following carboxylation of **C** provides rhodium benzoate Rh(O_2_CPh)(dcype) **D**. There are two possible pathways in the next step. Transmetallation of **D** and chloroaluminum species converts the catalyst back to [RhCl(dcype)]_2_ **A**, or transmetallation of **D** and methylaluminum species directly gives RhMe(dcype) **A**. However, the generated **A** could be converted to the most thermodynamically stable **1** *via* further transmetallation because of the equilibrium between **1** and **A**.^24,25^ In addition, there is a branch in this reaction. **A** reacts with CO_2_ to give Rh(OAc)(dcype) **E** in the same manner as **C**. Therefore, this reaction provides a mixture of acetic acid and benzoic acid.

The undesired formation of **E** should be operative if the reaction mixture contains a sufficient amount of CO_2_ in solution. Nevertheless, the desired C–H bond activation takes place because the true concentration of CO_2_ in solution is much lower than saturation. Importantly, the catalytic reaction did not work well in the absence of the chloride source. This is probably because the tricoordinated active species, RhMe(dcype) **A** was too unstable to be present at a higher concentration and decomposition of the Rh species occurred. The presence of chloride species keeps the resting state of the rhodium species as [RhCl(dcype)]_2_ **1**, which has a stable tetracoordinated dimeric structure and moderately reactive to transmetallation with the Al–Me species. This equilibrium limited the concentration of the active species, RhMe(dcype) **A**, and suppressed its decomposition. Transmetallation steps have attracted less interest in mechanistic studies, and their possible equilibria are mostly ignored. Our observation should be an interesting example to show the importance of the transmetallation equilibria in the catalytic cycle.

## Conclusions

This article describes a detailed mechanistic analysis of the rhodium-catalyzed carboxylation of simple aromatic compounds. Although no active intermediates were observable at all in this reaction, the proposed mechanism was mostly supported by several experiments. Most importantly, elucidation of the active species was achieved by designing appropriate precursors, and carrying out kinetic studies. 14-Electron rhodium complexes were found to be the key intermediates in both the C–H bond activation and carboxylation steps. The presence of such species was also supported by the formation of methane and acetic acid. KIE studies under catalytic conditions revealed that the C–H bond activation step was the turnover-limiting step. According to kinetic studies, this C–H bond activation step should be a minor pathway in the presence of a sufficient amount of CO_2_ because of undesired predominant carboxylation of the Rh^I^–Me species. However, further analysis revealed that this problem could be overcome to some extent by mechanical factors such as stirring rate and the shape of the reaction vessel, because undesired carboxylation of the Rh^I^–Me species could be made slower by controlling the concentration of CO_2_ in solution. Finally, it was found that reversible transmetallation pathways to give [RhCl(dcype)]_2_ **1** contributed to suppress decomposition of the catalyst. This study will provide new possibilities in such C–H bond functionalization reactions using alkyl-metal species and transition metal-catalyzed carboxylation reactions.

## References

[cit1] (i) Takaya J. and Iwasawa N., Science of Synthesis, C-1 Building Blocks in Organic Synthesis, 2014, vol. 1, pp. 281–307.

[cit2] Suga T., Mizuno H., Takaya J., Iwasawa N. (2014). Chem. Commun..

[cit3] Boogaerts I. I. F., Nolan S. P. (2010). J. Am. Chem. Soc..

[cit4] AlMe_1.5_(OEt)_1.5_ was prepared from AlMe_3_ with 2 equiv. of EtOH. The 1 : 1 sharp peaks of the methyl group and ethoxy group indicate this composition and its discrete structure. A tetramer structure has been postulated for the related compound. See: Turunen J. Pakkanen T. T. Löfgren B., J. Mol. Catal., 1997, 123 , 35 –42 , and ref. 2 .

[cit5] Price R. T., Andersen R. A., Muetterties E. L. (1989). J. Organomet. Chem..

[cit6] Kolomnikov I. S., Gusev A. O., Belopotapova T. S., Grigoryan M. K., Lysyak T. V., Struchkov Y. T., Vol'pin M. E. (1974). J. Organomet. Chem..

[cit7] Yang L., Huang H. (2015). Chem. Rev..

[cit8] Mizuno H., Takaya J., Iwasawa N. (2011). J. Am. Chem. Soc..

[cit9] Gao K., Yoshikai N. (2012). Chem. Commun..

[cit10] Zhou B., Hu Y., Wang C. (2015). Angew. Chem., Int. Ed..

[cit11] Fukumoto Y., Sawada K., Hagihara M., Chatani N., Murai S. (2002). Angew. Chem., Int. Ed..

[cit12] Ostapowicz T. G., Hölscher M., Leitner W. (2011). Chem.–Eur. J..

[cit13] Urtel H., Meier C., Eisenträger F., Rominger F., Joschek J. P., Hofmann P. (2001). Angew. Chem., Int. Ed..

[cit14] Hayashi T., Takahashi M., Takaya Y., Ogasawara M. (2002). J. Am. Chem. Soc..

[cit15] Hofmann P., Meier C., Hiller W., Heckel M., Riede J., Schmidt M. U. (1995). J. Organomet. Chem..

[cit16] Considered from this stabilization effect of PCy_3_, the role of DMA under catalytic conditions might be suppression of the catalyst decomposition. Presumably, it weakly coordinates to the vacant site of unstable 14-electron complexes

[cit17] Choi J.-C., Sakakura T. (2003). J. Am. Chem. Soc..

[cit18] GC analysis of the liquid phase revealed the formation of a very small amount of toluene (0.002 mmol, TON = 0.2), which might be generated by undesired reductive elimination from Rh(H)(Me)(Ph)(dcype) **B**

[cit19] Insufficient introduction of CO_2_ led to irreproducible results. See ESI for the practical experimental procedure

[cit20] According to ^31^P NMR analysis, the reaction mixture included a small amount of Rh(OAc)(dcype) **E** at lower concentrations of PCy_3_ and this affected the concentration of free PCy_3_. But this was trivial, so that linear correlation was mostly maintained throughout the experiment

[cit21] It should be noted that we used Chemistation™ (Tokyo Rikakikai Co. Ltd.) for heating a reaction mixture in other experiments including Scheme 9. This apparatus cools the system just above the liquid phase to maintain gentle reflux. On the other hand, experiments in Tables 1 and 2 were carried out in an oil bath, which does not have a cooling system for practical reasons. Such small differences also affected the results. For example, the TONs of BzOH and AcOH were 15 and 54 respectively, when the reaction in entry 1 (Table 1) was carried out using Chemistation™

[cit22] A decrease in CO_2_ should affect the results over longer reaction times. The amount of acetic acid in Scheme 9 (TON = 60) suggests that not as much acetic acid forms during the later stages of the reaction

[cit23] The experiments at higher temperatures were unsuccessful owing to the sensitivity of our equipment

[cit24] Addition of an appropriate ligand enables the observation of a methylrhodium(i) complex in such Rh–Cl/Al–Me transmetallation equilibrium. For example, the reaction of RhCl(PCy_3_)(dcype) **F** with AlMe_3_ gave RhMe(PCy_3_)(dcype) **2** as a major product: 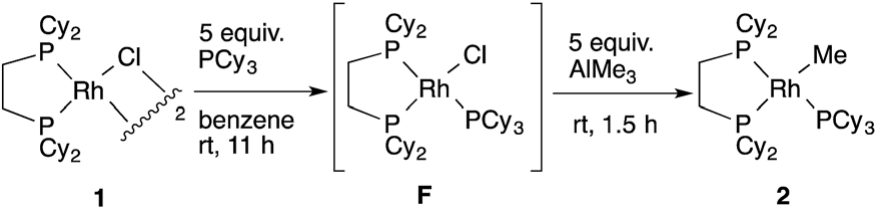 Interestingly, the use of AlMe_1.5_(OEt)_1.5_ resulted in no reaction under the similar conditions. These results might be related to the better results obtained using the latter reagent in the carboxylation reaction (ref. 2)

[cit25] There would also be an equilibrium between **1** and RhPh(dcype) **C** in a similar manner

