# Supplementary material for: Mechanistic study of the rhodium-catalyzed carboxylation of simple aromatic compounds with carbon dioxide
Source: Chem Sci. 2016 Nov 1;8(2):1454–62. doi: 10.1039/c6sc03838g (PMC5460598; doi:10.1039/c6sc03838g)

Electronic Supplementary Material (ESI) for Chemical Science.
This journal is © The Royal Society of Chemistry 2016

Mechanistic Study of Rhodium-Catalyzed Carboxylation of Simple Aromatic Compounds with Carbon Dioxide

Takuya Suga, Jun Takaya and Nobuharu Iwasawa*
Department of Chemistry, Tokyo Institute of Technology, O-okayama, Meguro-ku, Tokyo 152-8551, Japan
e-mail: niwasawa@chem.titech.ac.jp

Table of Contents

1. General Information ...S1
2. Procedure for The Preparation of Benzoic Acid Derivatives S1
3. Preparation of Rhodium Complexes **2**, **3**, **D**, **D'**, **E**, **E'** S2
4. ^{31}P NMR Analysis of The Reaction of RhMe(PCy_3)(dcype) **2** with Benzene And CO_2 ....S8
5. Kinetic Study of The Reaction of RhMe(PCy_3)(dcype) **2** with Benzene S9
6. Observation of Methane under Catalytic Conditions Using GC/FID S13
7. Procedure for The KIE Experiment under Catalytic Conditions S14
8. Kinetic Study of The Reaction of RhPh(PCy_3)(dcype) **3** with CO_2 ....S14
9. The Stoichiometric Reaction of **2** under CO_2 at 85 ºC S19
10. The Catalytic Carboxylation Reactions in Various Vessels S20
11. The *in situ*-IR Spectroscopy Experiment for The Observation of CO_2 in Toluene S20
12. Analysis of a Reaction Mixture by ^{31}P NMR S21
13. The Reaction of Rh(O_2CPh)(dcype) **D** with $AlMe_{1.5}(OEt)_{1.5}$ S21
14. The Reaction of Rh(O_2CPh)(dcype) **D** with $AlMe_{1.5}(OEt)_{1.5}$ in The Presence of $AlClMe_2$ S21
15. The Catalytic Carboxylation of Benzene Using Rh(O_2CPh)(dcype) **D** S22
16. Methylation of RhCl(PCy_3)(dcype) **F** with $AlMe_3$ S22
17. References S23
18. NMR Spectra of The New Compounds S24

1. General Information

All operations were performed under an argon atmosphere. ^{1}H, ^{13}C, ^{31}P NMR spectra were recorded on JEOL ECX-500 (500MHz for ^{1}H, 125 MHz for ^{13}C, and 200 MHz for ^{31}P) or Bruker DRX-500 (125 MHz for ^{13}C) using C_6D_6 (^{1}H, δ = 7.15; ^{13}C, δ = 128.0) and H_3PO_4 (^{31}P, δ = 0.0) as internal standards. Mass spectra were recorded on a JEOL JMS-700. Elemental analyses were performed on an elementar vario MICRO. GC chromatograms were recorded on a SHIMADSU GC-2010 equipped with DB-1 column (for detection of methane, toluene) or DB-WAXETR column (for detection of acetic acid). Wakogel B-5F coated on glass in a thickness of 0.9 mm was used for preparative TLC. All the NMR experiments were carried out using NMR tube with Young's Teflon bulb equipment. Benzene-d_6 and toluene-d_8 were dried and deoxygenated by treatment with sodium and benzophenone, followed by distillation under nitrogen atmosphere. The distillates were stored over molecular sieves 4A pellets in the glovebox. All the other liquid materials were distilled, degassed by argon bubbling, and stored in the glovebox.

Dehydrated dimethylacetamide (DMA), 1,2-bis(dicyclohexylphosphino)ethane (dcype), toluene-d_8 were purchased from Sigma-Aldrich co., LLC. Tetramethylurea (TMU) was purchased from Tokyo Chemical Industry co. LTD. Benzene was purchased from Wako Chemical Pure Industries, LTD. Benzene-d_6 was purchased from Kanto Chemical, co. INC.

$[RhCl(coe)_2]_2$, $[RhCl(cod)]_2$ and $[Rh(OAc)(cod)]_2$ were prepared by the known procedure.[i] $AlMe_{1.5}(OEt)_{1.5}$ and $[RhCl(dcype)]_2$ **1** were prepared as we previously reported.[ii]

2. Procedure for The Preparation of Benzoic acid derivatives: A Typical Procedure Was Described for The Reaction of Benzene.

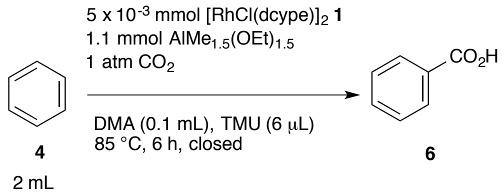

A solution of $[RhCl(dcype)]_2$ **1** (5.6 mg, 0.005 mmol), DMA (0.1 mL), TMU (6 μL, 0.05 mmol) and $AlMe_{1.5}(OEt)_{1.5}$ (132 mg, 1.1 mmol) in benzene (2 mL) was stirred in a glass tube (φ = 2.0 cm, 18 cm) at room temperature for a few minutes, and then the system was purged with an atomospheric pressure of CO_2. The mixture was heated at 85 °C (outer temperature) for 6 h in the closed system. 2N HCl aq. and diethyl ether were added and the mixture was vigorously stirred for a few minutes. The mixture was filtered through a short plug of cotton, and the filtrate was extracted with diethyl ether three times. The combined organic layer was extracted with NaOH aq. three times

and the combined aqueous layer was acidified with HCl aq., then extracted with diethyl ether three times, and the extract was dried over $MgSO_4$. After removal of solvent under reduced pressure, the residue was purified by silica-gel column chromatography (CH_2Cl_2 : AcOEt = 9 : 1) to give benzoic acid as a white solid (44.5 mg, 0.37 mmol).

In several experiments, the methods shown below were employed instead to quantify acetic acid or toluene.

Acetic acid (**Scheme 9**): 1N HCl (2 mL) and diethyl ether (4 mL) were added to the reaction mixture after heating under CO_2, and the mixture was stirred for 30 minutes at room temperature. Dodecane (internal standard) was added to the resulting mixture, which was diluted with methanol until it became homogeneous, and was analyzed by GC to quantify acetic acid.

Toluene (ref. 17): The extract was analyzed by GC before concentration to quantify toluene, using dodecane as an internal standard.

3. Preparation of Rhodium Complexes

Synthesis of RhMe(PCy_3)(dcype) 2 (Scheme 3)

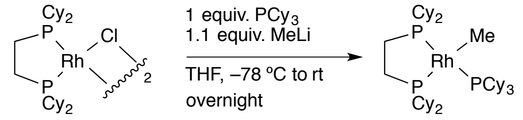

1 **2**

To a 100 mL two-necked flask were added $[RhCl(dcype)]_2$ **1** (561 mg, 1.0 mmol), tricyclohexylphosphine (280 mg, 1.0 mmol) and THF (25 mL). After the mixture was cooled to –78 ºC, MeLi (1.1 M in diethyl ether, 1.1 mmol, 1.0 mL) was added dropwise to the mixture, which was stirred overnight at room temperature. In the glovebox, the precipitate was collected by filtration, washed with THF, and was dried *in vacuo* to give RhMe(PCy_3)(dcype) **2** as a yellow powder (482 mg, 0.57 mmol, 57 %).

^{1}H-NMR (500 MHz, C_6D_6) δ 2.51–2.59 (br, 2H), 2.34–2.46 (br, 5H), 2.13–2.28 (br, 8H),1.60–1.92 (m, 32H), 1.13–1.57 (m, 34H), 0.35–0.43 (m, 3H); ^{13}C-NMR (125 MHz, C_6D_6) δ 39.5 (d, J = 9.4 Hz), 36.1 (d, J = 19.1 Hz), 32.0 (d, J = 7.5 Hz), 31.2, 31.0, 29.9 (d, 14.5 Hz), 28.8 (d, J = 7.1 Hz) 28.3–27.7 (m), 27.5, 27.2 (d, J = 3.0 Hz), 25.5–25.1 (m), 23.3–22.9 (m), -7.5–-8.5 (m), two peaks overlap; ^{31}P-NMR (200 MHz, C_6D_6) δ 76.4 (ddd, $J_{Rh\text{-}P}$ = 159 Hz, $J_{P\text{-}P}$ = 321, 22 Hz), 67.0 (ddd, $J_{Rh\text{-}P}$ = 124 Hz, $J_{P\text{-}P}$ = 22, 22 Hz), 33.4 (ddd, $J_{Rh\text{-}P}$ = 138 Hz, $J_{P\text{-}P}$ = 321, 22 Hz), Anal. Calcd for $C_{45}H_{84}P_3Rh$: C, 65.84; H, 10.32; Found: C, 65.82; H, 9.85.

Synthesis of RhPh(PCy$_3$)(dcype) 3 (Scheme 3)

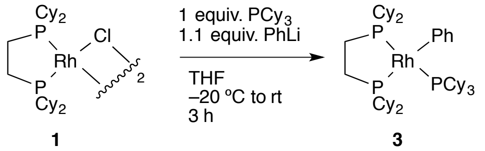

In a glovebox, to a 100 mL two-necked flask were added [RhCl(dcype)]$_2$ **1** (449 mg, 0.80 mmol), tricyclohexylphosphine (224 mg, 0.80 mmol) and THF (30 mL) and the mixture was cooled to –35 ºC. A THF (5 mL) solution of PhLi (74 mg, 0.88 mmol) was added dropwise to the mixture, which was stirred for 1 h, and then the mixture was concentrated *in vacuo*. Toluene was added to the residue to separate LiCl as a precipitate, and the mixture was filtered through Celite. After concentration of the filtrate, the residue was suspended in ca. 10 mL of THF to dissolve impurities. The mixture was stirred for a few minutes, and then stored overnight in a refrigerator. The precipitated solid was collected by filtration and dried *in vacuo* to give RhPh(PCy$_3$)(dcype) **3** as a yellow powder (313 mg, 0.35 mmol, 44 %). A single crystal suitable for X-ray analysis was prepared from THF/DMA (Figure S1).

^{1}H-NMR (500 MHz, C_6D_6) δ 7.97 (m, 2H), 7.18 (t, J =7.1 Hz, 2H), 7.02 (t, J = 7.1 Hz, 1H), 2.48–2.66 (br, 2H), 2.24–2.42 (br, 2H); 1.97–2.17 (m, 8H), 1.52–1.97 (m, 35H), 1.00–1.52 (m, 34H), 0.74–0.94 (m, 2H); ^{13}C-NMR (125 MHz, C_6D_6) δ 174.9–173.8 (m), 142.2, 125.3, 120.1, 38.7 (d, J = 9.5 Hz), 38.1 (br), 36.6 (d, J = 20 Hz), 32.1 (d, J = 7.2 Hz) 31.7 (d, J = 11.9 Hz), 31.1, 29.8 (d, J = 15.5 Hz), 29.1, 28.7–27.8 (m), 27.3, 27.1, 26.9, 24.6–23.9 (m), one peak overlaps; ^{31}P-NMR (200 MHz, C_6D_6) δ 65.7 (ddd, $J_{Rh\text{-}P}$ = 162 Hz, $J_{P\text{-}P}$ = 296, 22 Hz), 65.3 (ddd, $J_{Rh\text{-}P}$ = 113 Hz, $J_{P\text{-}P}$ = 22, 22 Hz), 26.3 (ddd, $J_{Rh\text{-}P}$ = 145 Hz, $J_{P\text{-}P}$ = 296, 22 Hz); Anal. Calcd for $C_{50}H_{86}P_3Rh$: C, 67.96; H, 9.86; Found: C, 68.42; H, 9.51.

Figure S1. The crystal structure of **3**.

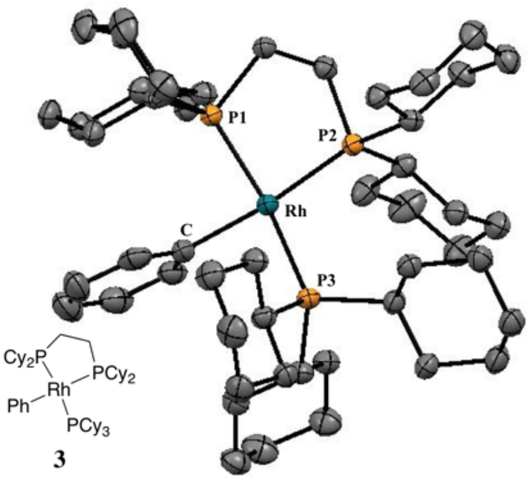

ORTEP diagrams of **3** (CCDC 1017905). Thermal ellipsoids are shown at the 50% probability level; H atoms and solvent molecules have been omitted for clarity. Selected bond lengths (Å) and angles (deg): Rh-C, 2.079(4); Rh-P1, 2.2771(8); Rh-P2, 2.2824(9); Rh-P3, 2.3685(8); P1-Rh-P2, 85.93(3); P3-Rh-C, 88.4(1), P1-Rh-P3, 161.04(3); P2-Rh-C, 160.3(1).

Preparation of Rh(O_2CPh)(dcype) D

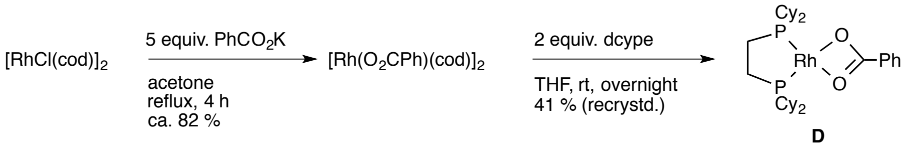

• Synthesis of [Rh(O_2CPh)(cod)]$_2$

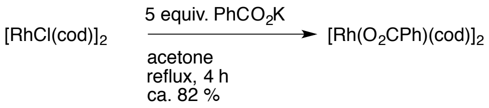

To a 100 mL round-bottom flask were added powdered KOH (>85 % purity, 313 mg, > 4.9 mmol), EtOH (10 mL) and benzoic acid (610 mg, 5.0 mmol), and the mixture was stirred for several minutes. After the solvent was removed *in vacuo*, [RhCl(cod)]$_2$ (493 mg, 1.0 mmol) and acetone (15 mL) were added. A Dimroth condenser and a balloon were attached to the vessel, which was purged with nitrogen. The mixture was refluxed for 4 h to give an orange suspension, which was dissolved with CH_2Cl_2, and then filtered through Celite. The filtrate was concentrated, washed with acetone and water to give [Rh(O_2CPh)(cod)]$_2$ in 82 % yield (548 mg, 0.83 mmol) as an orange powder. This material was used in the next step without further purification.

^{1}H NMR (500 MHz, C_6D_6) δ 8.03 (dd, J = 8.4, 1.4 Hz, 2H), 7.03-6.95 (m, 3H), 4.56-4.16 (broad, 4H), 2.86-2.28 (broad, 4H), 1.74-1.50 (broad, 4H).

• Synthesis of Rh(O_2CPh)(dcype) D

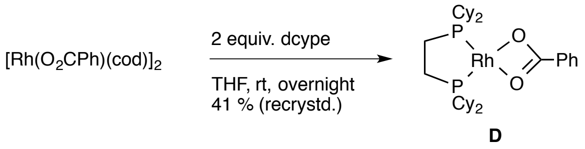

In a glovebox, a solution of 1,2-bis(dicyclohexylphosphino)ethane (211 mg, 0.5 mmol) in THF (20 mL) was added dropwise to a solution of [Rh(O_2CPh)(cod)]$_2$ (166 mg, 0.25 mmol) in THF (30 mL) and the mixture was stirred overnight at room temperature. The mixture was concentrated *in vacuo* and dissolved again in toluene. After the insoluble residue was filtered off through Celite, the filtrate was concentrated, and the residue was recrystallized from toluene/pentane in a refrigerator to give **D** in 41 % yield (134 mg, 0.21 mmol) as an orange powder.

^{1}H-NMR (500 MHz, C_6D_6) δ 8.44-8.39 (m, 2H), 7.04-6.98 (m, 3H), 2.41 (d, J = 11.5 Hz, 4H), 1.90-1.52 (m, 28H), 1.30-1.10 (m, 12H), 1.04 (d, J = 11.5 Hz, 4H); ^{13}C-NMR (125 MHz, C_6D_6) δ 184.3, 135.8, 131.3, 129.4, 36.9 (t, J = 11.9 Hz), 29.8 (d, J = 44.1 Hz), 27.5 (d, J = 3.6 Hz), 26.6, 21.9 (t, J = 23.9 Hz), one peak overlaps; ^{31}P-NMR (200 MHz, C_6D_6) δ 103.6 (d, J = 199 Hz); HR-MS (FAB$^+$): Calcd for $C_{33}H_{54}O_2P_2$Rh $[M+1]^+$: 647.2649; Found: 647.2690.

Observation of Rh(O_2CPh)(PCy_3)(dcype) D'

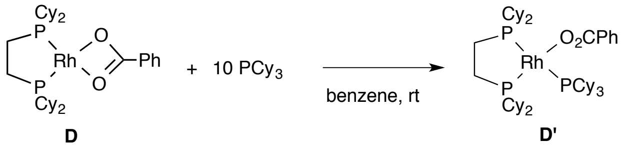

In a glovebox, to an NMR tube was added Rh(O_2CPh)(dcype) **D** (3.2 mg, 0.005 mmol), PCy_3 (14.0 mg, 0.05 mmol) and benzene (0.5 mL) at room temperature and the mixture was stood for 1.5 h. Formation of a new complex assignable as Rh(O_2CPh)(PCy_3)(dcype) **D'** was observed by ^{31}P NMR.

^{31}P-NMR (200 MHz, C_6D_6) δ 84.7 (ddd, $J_{Rh\text{-}P}$ = 183 Hz, $J_{P\text{-}P}$ = 30, 30 Hz), 77.9 (ddd, $J_{Rh\text{-}P}$ = 152 Hz, $J_{P\text{-}P}$ = 304, 30 Hz), 28.0 (ddd, $J_{Rh\text{-}P}$ = 127 Hz, $J_{P\text{-}P}$ = 304, 30 Hz).

Figure S2. ^{31}P NMR spectrum of $Rh(O_2CPh)(PCy_3)(dcype)$ **D'.**

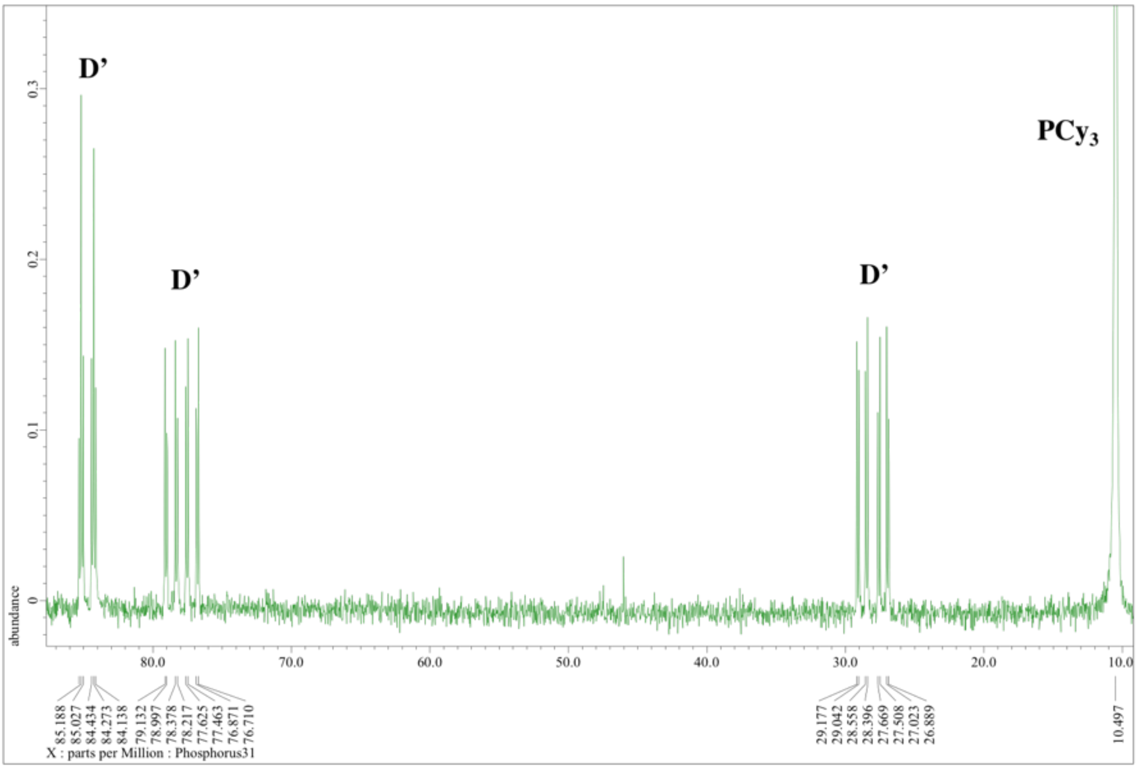

<u>Synthesis of Rh(OAc)(dcype) E</u>

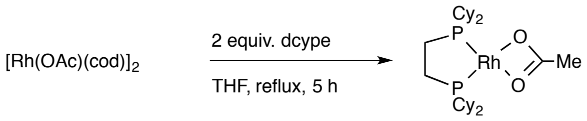

E

In a glovebox, a solution of 1,2-bis(dicyclohexylphosphino)ethane (1.69 g, 4.0 mmol) in THF (25 mL) was added dropwise to a solution of $[Rh(OAc)(cod)]_2$ (1.08 g, 2.0 mmol) in THF (25 mL) and the mixture was refluxed for 5 h. The mixture was concentrated *in vacuo* and the residue was recrystallized from THF/pentane in a refrigerator to give **E** in 56 % yield (1.31 g, 2.24 mmol) as a yellow powder.[iii]

^{1}H-NMR (500 MHz, C_6D_6) δ 2.34 (d, J = 11.0 Hz, 4H), 1.85-1.93 (s, 3H), 1.50-1.85 (m, 28H), 1.11-1.24 (m, 12H), 1.00 (d, 4H, J = 10.5 Hz, 4H); ^{13}C-NMR (125 MHz, C_6D_6) δ 189.8, 36.9 (t, J =11.9 Hz), 29.8 (d, J = 32.2 Hz), 27.4–27.6 (m), 26.6, 24.9, 21.9 (td, J = 21.5, 4.8 Hz); ^{31}P-NMR (200 MHz, C_6D_6) δ 103.4 (d, J = 194 Hz); HR-MS (FAB$^+$): Calcd for $C_{28}H_{51}O_2P_2Rh$ $[M]^+$: 584.2419; Found: 584.2438.

Observation of Rh(OAc)(PCy_3)(dcype) E'

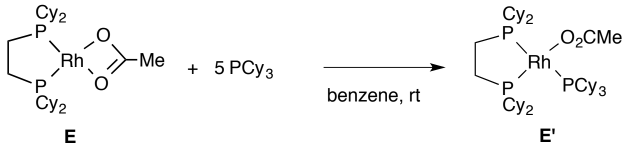

In a glovebox, to an NMR tube was added Rh(OAc)(dcype) **E** (5.8 mg, 0.01 mmol), PCy_3 (14.0 mg, 0.05 mmol) and benzene (0.5 mL) at room temperature and the mixture was stood for 1.5 h. Formation of a new compound assignable as Rh(OAc)(PCy_3)(dcype) **E'** was observed by ^{31}P NMR. ^{31}P-NMR (200 MHz, C_6D_6) δ 84.4 (ddd, $J_{Rh\text{-}P}$ = 183 Hz, $J_{P\text{-}P}$ = 31, 31 Hz), 77.9 (ddd, $J_{Rh\text{-}P}$ = 150 Hz, $J_{P\text{-}P}$ = 308, 31 Hz), 28.3 (ddd, $J_{Rh\text{-}P}$ = 127 Hz, $J_{P\text{-}P}$ = 308, 31 Hz).

Figure S3. ^{31}P NMR spectrum of Rh(OAc)(PCy_3)(dcype) **E'**.

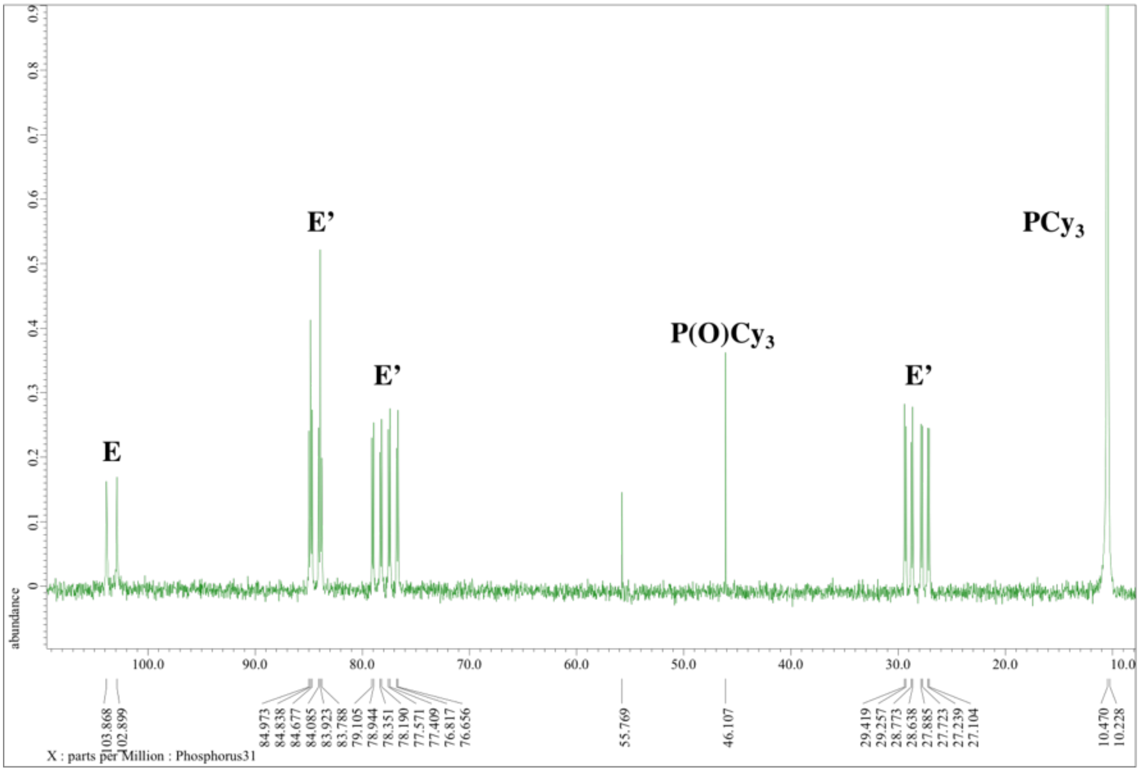

4. ^{31}P NMR Analysis of The Reaction of RhMe(PCy$_3$)(dcype) 2 with Benzene and CO_2 (Scheme 4)

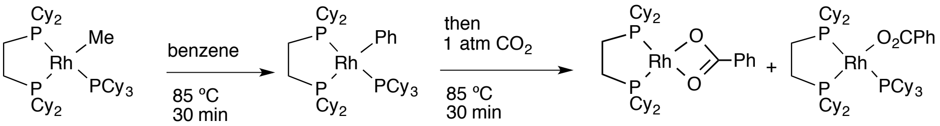

1 **2** **D** **D'**

In a glovebox, RhMe(PCy$_3$)(dcype) **2** (4.1 mg, 0.005 mmol) was suspended in benzene (0.5 mL). The mixture was transferred to an NMR tube with a sealed thin glass tube, which contained a benzene solution of PPh$_3$ as an internal standard. The mixture was heated at 85 ºC for a few seconds to dissolve **2** completely, and then the mixture was monitored by ^{31}P NMR. After heating at 85 ºC for 30 minutes, the peaks of **2** disappeared and phenylrhodium **3** was observed instead. Then, the NMR tube was purged with CO_2 and heated at 85 ºC for further 30 minutes. The formation of **D** and **D'** was confirmed by ^{31}P NMR. ^{31}P NMR spectra of observed **3**, **D** and **D'** were consistent with those of authentic samples prepared by the methods described above.

Figure S4. ^{31}P NMR spectra of the reaction of **2**.

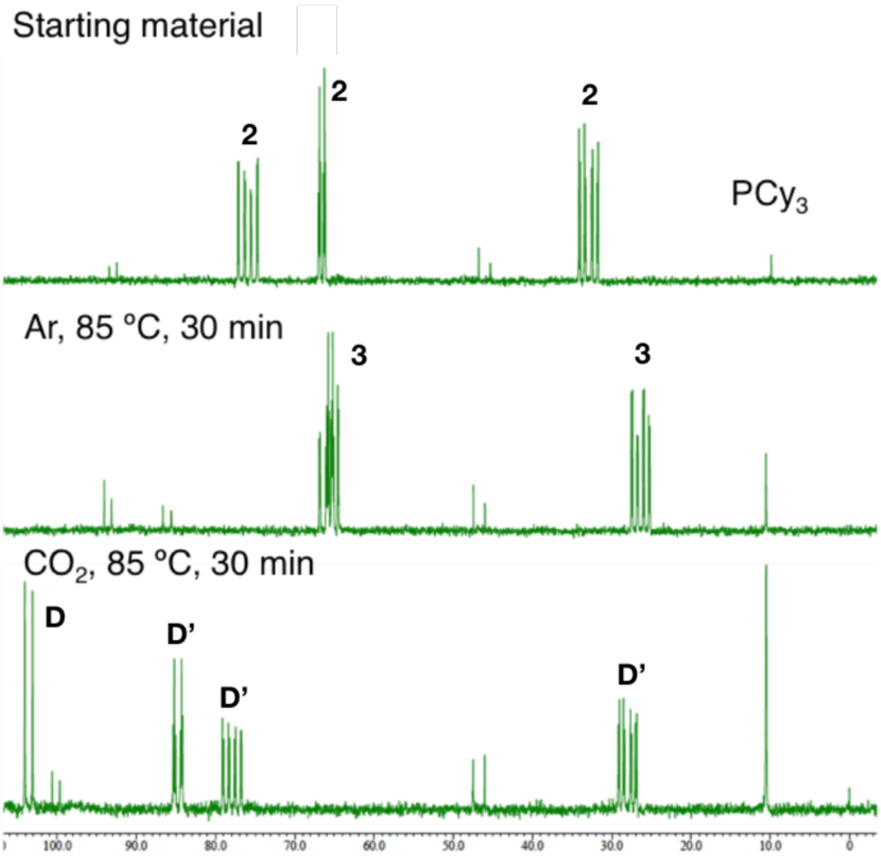

5. Kinetic Study of The Reaction of RhMe(PCy3)(dcype) 2 with Benzene (Figure 1)

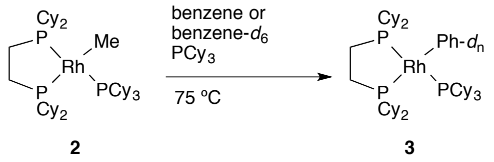

Figure S5. The apparatus for ^{1}H NMR study.

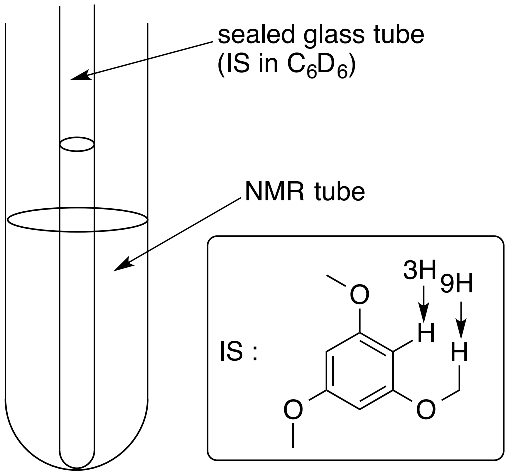

In a glovebox, a solution of PCy_3 in benzene was prepared by using volumetric flask. To an NMR tube were added 0.50 mL of this solution, RhMe(PCy_3)(dcype) **2** (4.1 mg, 0.005 mmol), and a sealed thin glass tube which contained a solution of 1,3,5-trimethoxybenzene (internal standard) in benzene (Figure S3). The mixture was heated to 75 ºC for a few seconds to dissolve **2** completely. Then, the reaction of **2** and benzene was monitored by ^{1}H NMR at 75 ºC. The spectra were recorded in every 5 minutes using noD–NMR. Consumption of **2** was monitored by comparing the intensity of the peaks of Rh–Me moiety (δ 0.3 ppm) with that of O–Me moiety of 1,3,5-trimethoxybenzene (δ 3.3 ppm). The gradual formation of methane (δ 0.13 ppm) was also detected along with the reaction (it could not be quantified due to its diffusion to the gas phase). Reactions were carried out using 0.025, 0.036, 0.050 and 0.10 M PCy_3 solutions in benzene. The reaction was first order to [**2**] and inverse first order to [PCy_3]. The linear correlation between –ln([**2**]/[**2**]$_0$) and the reaction time was maintained even after > 90 % consumption of **2** in the presence of 0.025 M PCy_3. Formation of RhPh(PCy_3)(dcype) **3** was ensured by ^{31}P NMR after the reaction. The rate equations and the rate constant are given in eq. S1.

$$-\ln\left(\frac{[\mathbf{2}]}{[\mathbf{2}]_0}\right) = k_{\mathrm{H}}[\mathrm{PCy_3}]^{-1}t, \quad k_{obs} = k_{\mathrm{H}}[\mathrm{PCy_3}]^{-1} \qquad \text{(S1)}$$

$$k_{\mathrm{H}} = 6.9 \times 10^{-4}\ [\mathrm{M \cdot min^{-1}}]\ \text{at 75 ºC}$$

The detailed results were summarized in Figure S6. The representative ^{1}H NMR spectra during the measurement and the ^{31}P NMR spectrum after the measurement were provided in Figure S7 and S8.

Figure S6. Kinetic study of C-H bond activation in benzene-d_0.

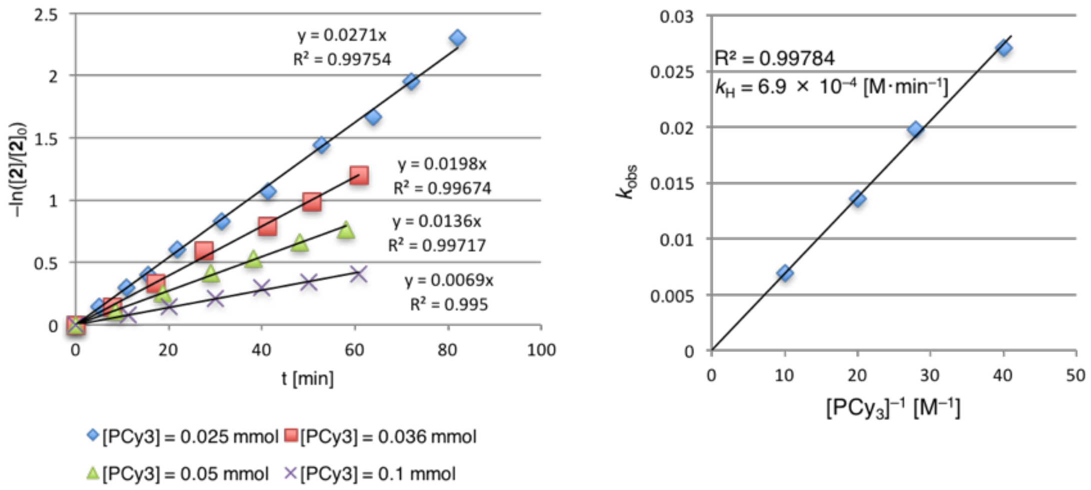

Figure S7. Kinetic study of C-H bond activation in benzene-d_0 by ^{1}H NMR. [PCy_3] = 0.050 M. The spectra are those recorded in every 20 minutes.

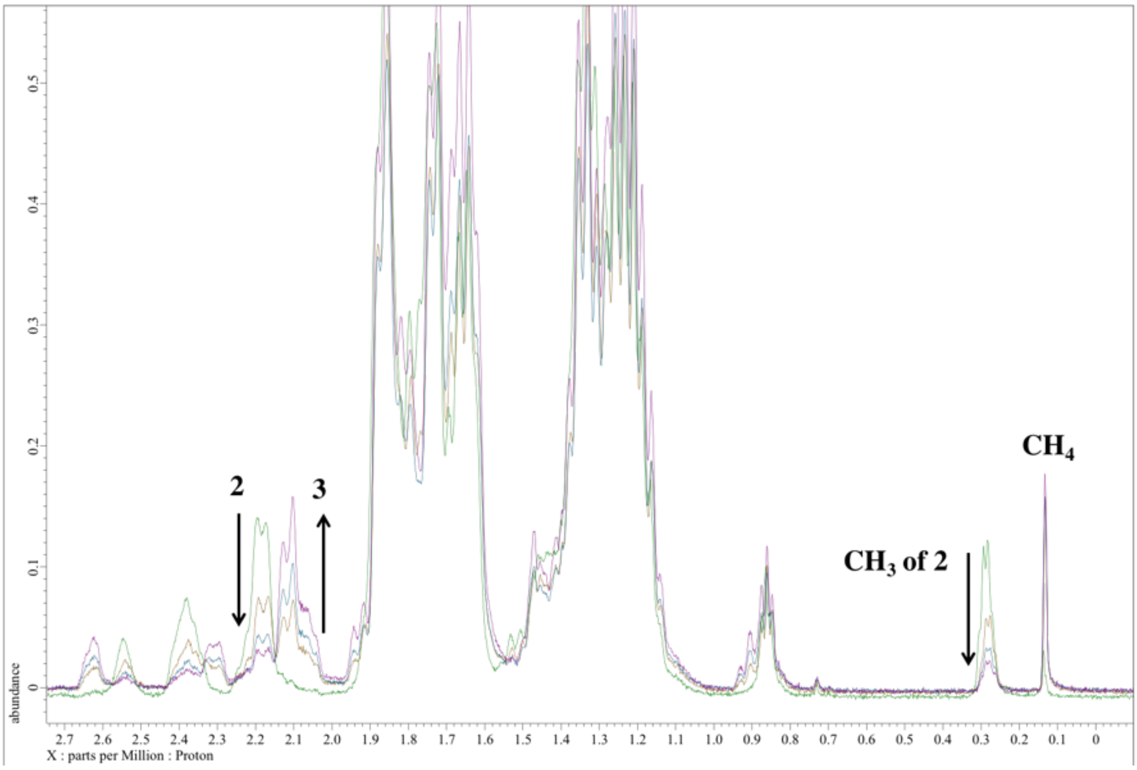

Figure S8. ^{31}P NMR spectrum after the kinetic study of C-H bond activation in benzene-d_0 (1.25 h, [PCy_3] = 0.050 M).

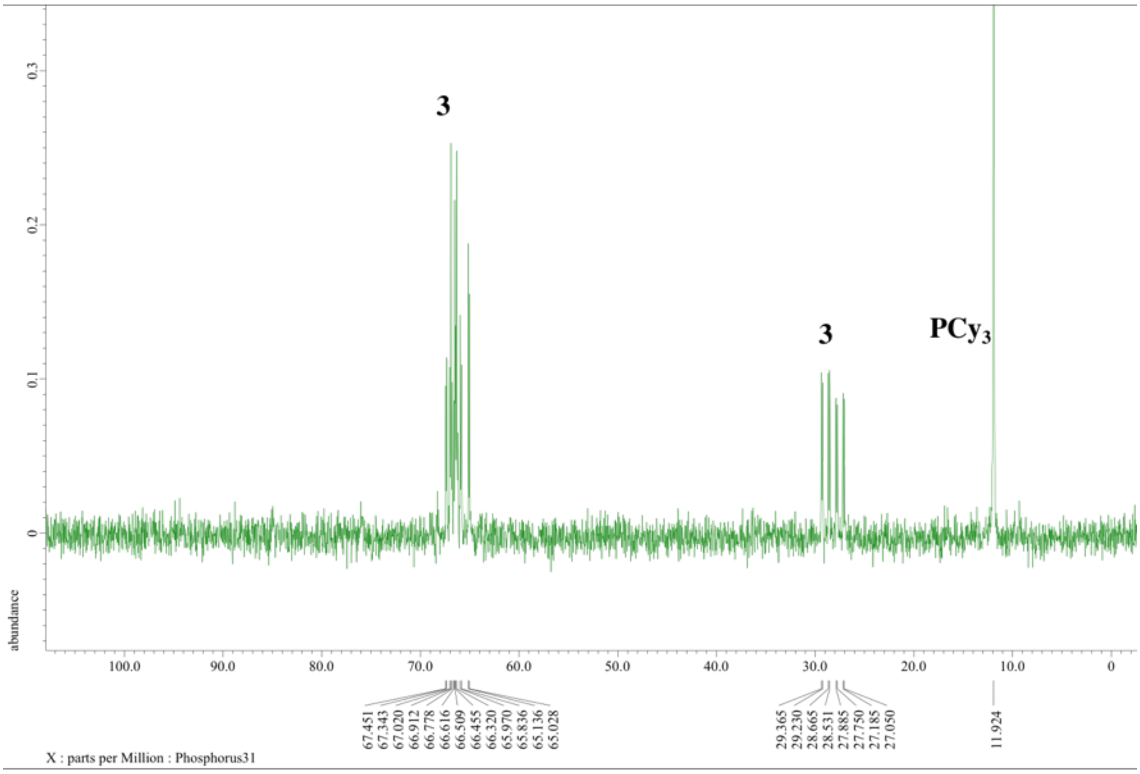

The same experiment was also carried out using benzene-d_6 instead of benzene (Figure 9). In this case, conventional ^{1}H NMR was used and the spectra were recorded in every 20 minutes. Methane-d_1 (δ 0.13 ppm, t, J = 1.8 Hz) was observed instead of methane. The reaction was first order to [**2**] and inverse first order to [PCy_3]. The rate equations and the rate constant are given in eq. S2.

$$-\ln\left(\frac{[\mathbf{2}]}{[\mathbf{2}]_0}\right) = k_{\mathrm{D}}[\mathrm{PCy_3}]^{-1}t, \quad k_{obs} = k_{\mathrm{D}}[\mathrm{PCy_3}]^{-1} \qquad \text{(S2)}$$

$$k_{\mathrm{D}} = 1.0 \times 10^{-4}\ [\mathrm{M} \cdot \mathrm{min}^{-1}]\ \text{ at 75 ºC}$$

The detailed results were summarized in Figure S9. The representative ^{1}H NMR spectra during the measurement were provided in Figure S10.

Figure S9. Kinetic study of C-H bond activation in benzene-d_6.

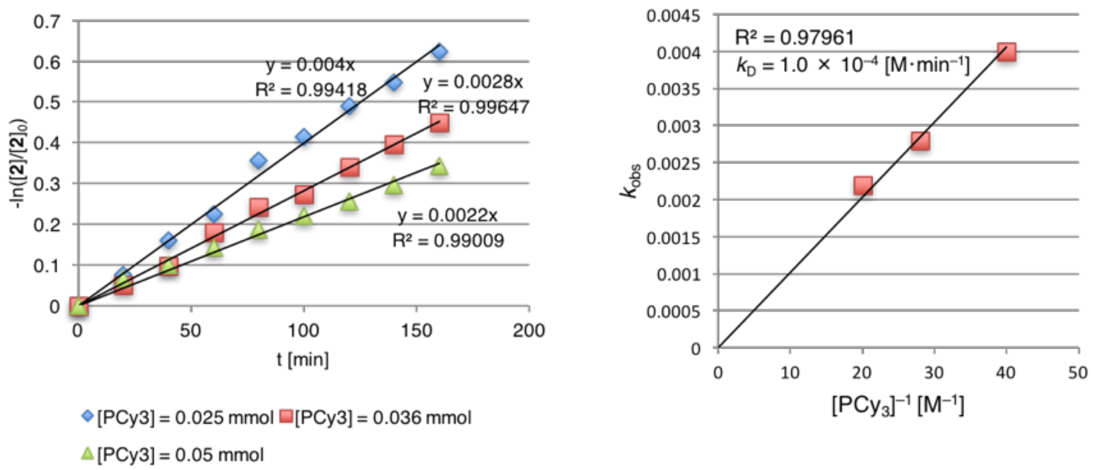

Figure S10. Kinetic study of C-H bond activation in benzene-d_6 by ^{1}H NMR. [PCy_3] = 0.0025 M. The shown spectra are those recorded in every 40 minutes. CH_3D was identified separately with higher resolution.

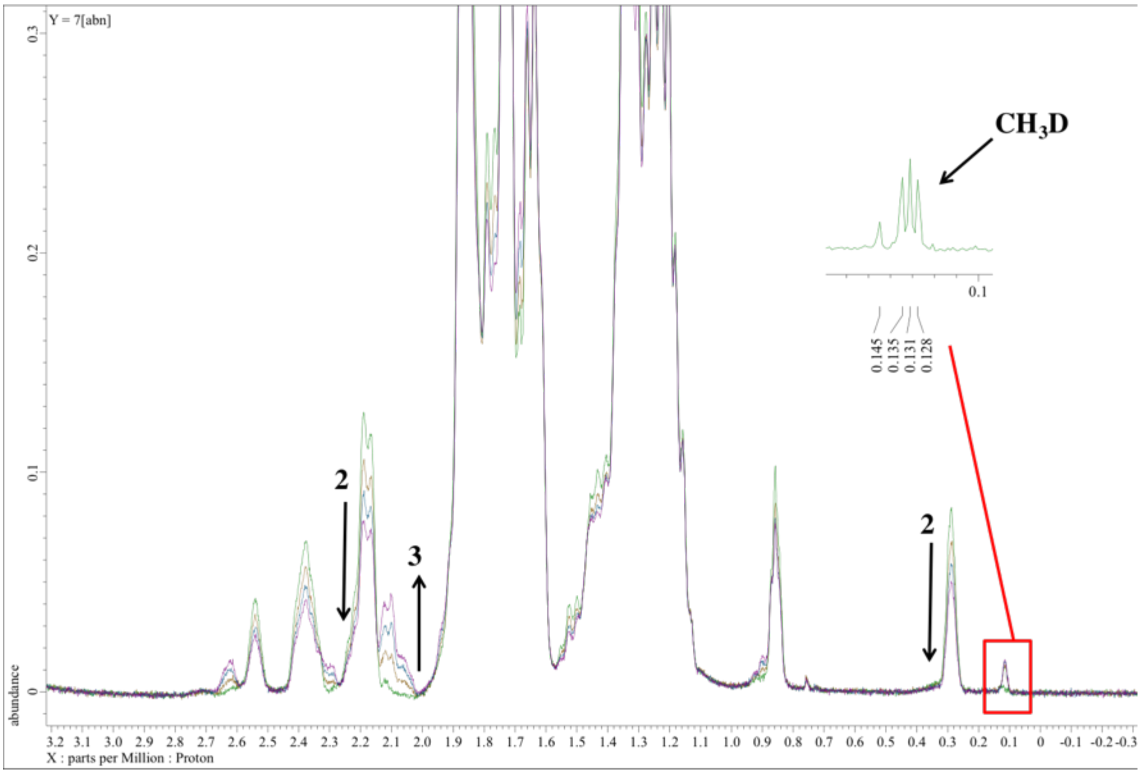

6. Observation of Methane under Catalytic Conditions Using GC/FID (Figure 2)

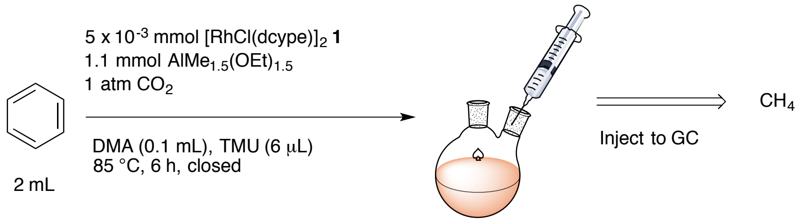

Conditions:

System	Shimadsu GC 2010
Column	DB-1 30m x 0.25mm
Column temp.	30 °C
Column flow	0.5-0.8 mL/min
Injection	Split flow
Detector	FID

After the catalytic carboxylation of benzene was carried out under the standard conditions, a part of the gas phase of the vessel was pulled out by a syringe and it was plunged into GC (Shimadzu 2010 A series, detailed conditions are described above) Its GC chromatogram indicated the presence of methane (Figure S11, blue). It was further confirmed by checking the GC chromatogram of the mixture of the gas phase and standard methane (black). The formation of ethane was ruled out by the similar procedure (brown). Almost no formation of methane was observed when the reaction was carried out in the absence of Rh catalyst (red).

Figure S11. The GC chromatograms of the gas phase after the reaction.

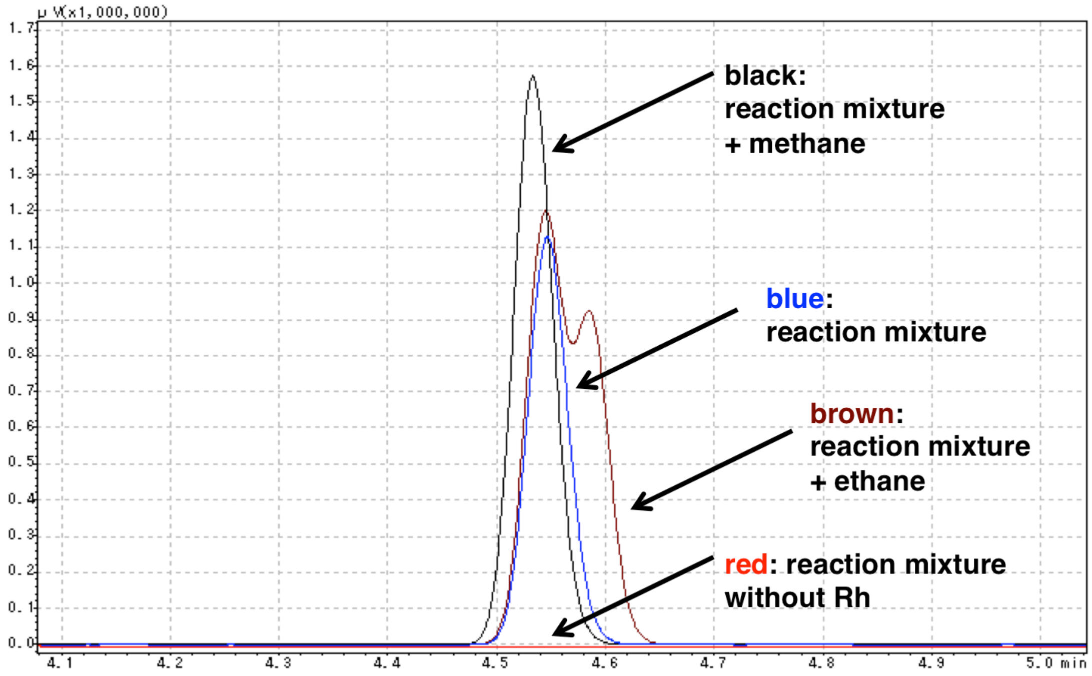

7. Procedure for The KIE Experiment under Catalytic Conditions (Scheme 6)

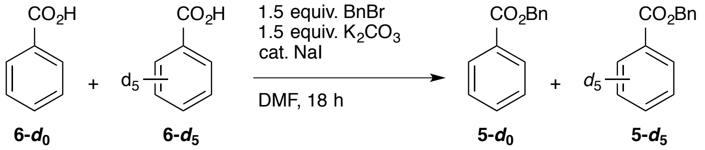

The KIE of the reaction between benzene-d_0 and benzene-d_6 was estimated by ^{1}H-NMR after esterification with benzyl bromide according to the known procedure.[iv] A mixture of benzoic acid **6-d_0** and benzoic acid-d_5 **6-d_5** was prepared according to the general procedure, except that the reaction time was 1 h and a mixed solution of each substrate (1 mL : 1 mL) was used.

To a 10 mL bial, a crude mixture of benzoic acid **6-d_0** and benzoic acid-d_5 **6-d_5** (which contained total of ca. 0.1 mmol of products based on weight), DMF (1 mL), K_2CO_3 (21 mg, 0.15 mmol) and NaI (6 mg, 0.04 mmol) were added at room temperature. After a few minutes, benzyl bromide (18 μL, 0.15 mmol) was added and the mixture was stirred for 18 h. The reaction mixture was diluted with diethyl ether, and then quenched with aquaous NH_4Cl. The organic layer was washed with water three times, dried over $MgSO_4$, and concentrated *in vacuo*. The crude mixture was purified by preparative TLC (hexane : ethyl acetate = 19 : 1) to give a mixture of benzyl benzoate **5-d_0** and benzyl benzoate-d_5 **5-d_5**. KIE ([**5-d_0**]/[**5-d_5**]) was calculated to be 5.5 on average of two sets of reactions by comparing integration of benzylic 2H (5.36 ppm) and 2H at ortho-positions of carboxy group (8.08 ppm) to those of authentic **5**. Authentic **5** was prepared as above from commercially available benzoic acid **6** (12.2 mg, 0.10 mmol). The yield was 80 % after purification.

8. Kinetic Study of The Reaction of RhPh(PCy$_3$)(dcype) 3 with CO_2 (Figure 3, 4)

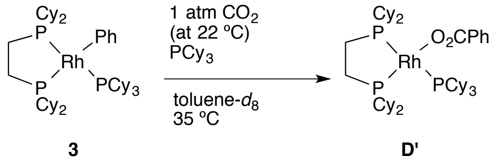

In a glovebox, a solution of PCy_3 in toluene-d_8 was prepared by using volumetric flask. To an NMR tube were added 0.50 mL of this solution, RhPh(PCy$_3$)(dcype) **3** (4.4 mg, 0.005 mmol), and a sealed thin glass tube which contained a solution of 1,3,5-trimethoxybenzene (internal standard) in toluene-d_8. The system was degassed three times by freeze-pump-thaw technique at –196 ºC, and then atmospheric pressure of CO_2 was introduced at –78 ºC. The NMR tube was gently shaken at this temperature for a few minutes to dissolve sufficient CO_2. Then, the sample was gradually warmed to room temperature (22 ºC) and was kept shaken for a few minutes. (*Note*: This operation must be carried out *under CO_2 flow* to keep internal pressure at 1 atm.) During this, an intensive

evolution of CO_2 bubble was observed, suggesting the liquid phase was saturated with CO_2. After the system was closed, it was stored at –78 ºC until starting to monitor the reaction. It was warmed again to room temperature being shaken just before the measurement. The reaction of **3** and CO_2 was monitored by ^{1}H NMR at 35 ºC. The spectra were recorded in every 5 minutes. Consumption of **3** was monitored by comparing the intensity of the peaks of aromatic C-H (*ortho* to Rh, δ 7.9 ppm) with that of aromatic C-H of 1,3,5-trimethoxybenzene (δ 6.2 ppm). The quantitative formation of a new compound, which had a characteristic peak at δ 8.4 ppm (2H, d, J = 6.5 Hz), was also observed. ^{31}P NMR spectra of the generated compound was consistent with those of Rh(O_2CPh)(PCy_3)(dcype) **D'**, which was prepared as described above. Reactions were carried out using 0.050, 0.072, 0.10, and 0.20 M PCy_3 solutions in toluene-d_8. The reaction was almost first order to [**3**] and inverse first order to [PCy_3]. The linear correlation between –ln([**3**]/[**3**]$_0$) and the reaction time was maintained even after > 90 % consumption of **3** in the presence of 0.050 M PCy_3. The rate equations and the rate constants are given in eq. S3.

$$-\ln\left(\frac{[\mathbf{3}]}{[\mathbf{3}]_0}\right) = (k_{\mathrm{Ph1}}[\mathrm{PCy_3}]^{-1} + k_{\mathrm{Ph2}})t, \quad k_{obs} = k_{\mathrm{Ph1}}[\mathrm{PCy_3}]^{-1} + k_{\mathrm{Ph2}} \qquad \text{(S3)}$$

$$k_{\mathrm{Ph1}} = 2.5 \times 10^{-3}\ [\mathrm{M \cdot min^{-1}}]$$

$$k_{\mathrm{Ph2}} = 8.0 \times 10^{-3}\ [min^{-1}]$$

$$\text{under } \mathrm{CO_2} \text{ (1 atm at 22 ºC) at 35 ºC}$$

The detailed results were summarized in Figure S12. The representative ^{1}H NMR spectra during the measurement and the ^{31}P NMR spectrum after the measurement were provided in Figure S13 and S14.

Figure S12. Kinetic study of carboxylation of **3** in toluene-d_8.

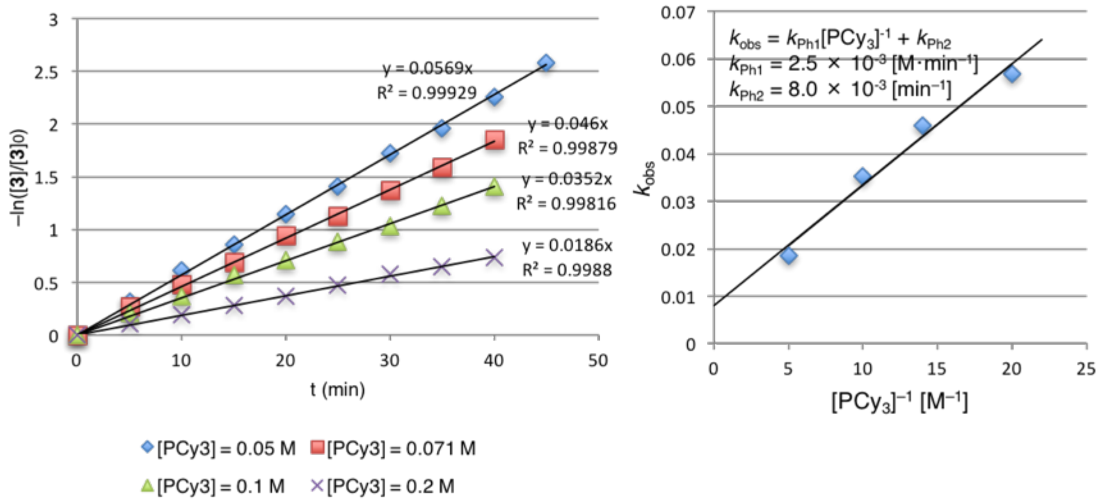

Figure S13. Kinetic study of carboxylation of **3** by ^{1}H NMR. [PCy_3] = 0.050 M. The spectra are those recorded in every 15 minutes.

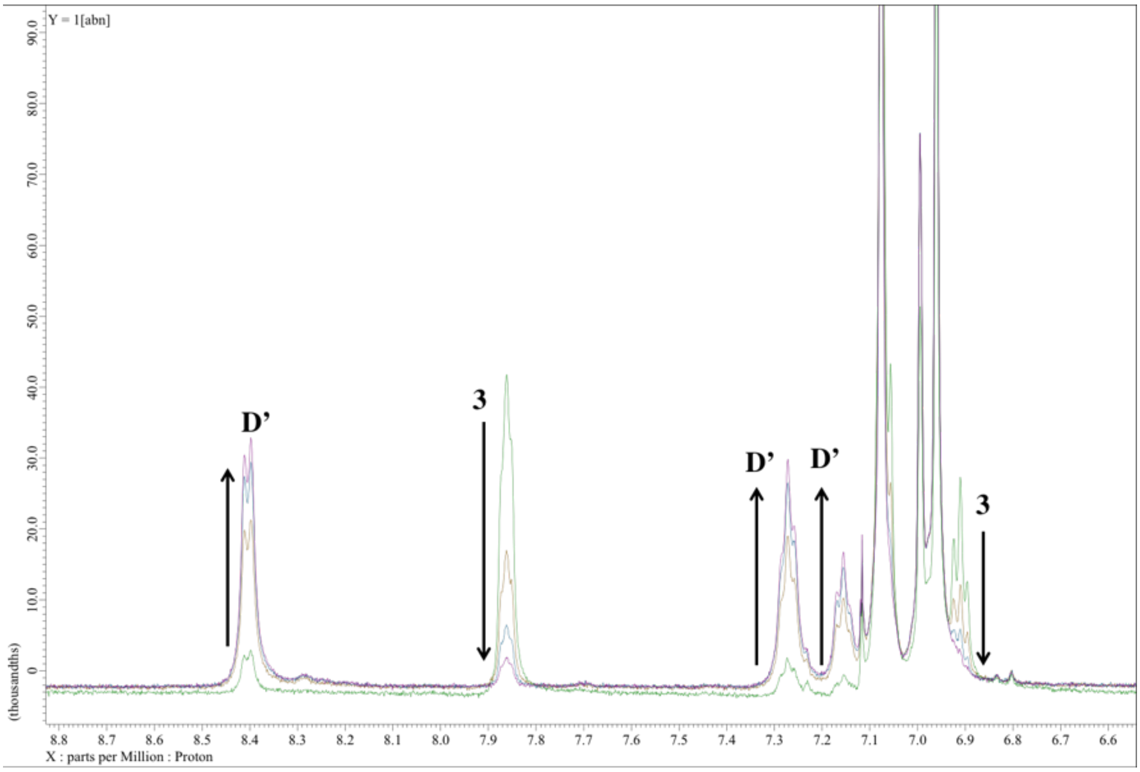

Figure S14. ^{31}P NMR spectrum after the kinetic study of carboxylation of **3** (50 min, [PCy_3] = 0.050 M).

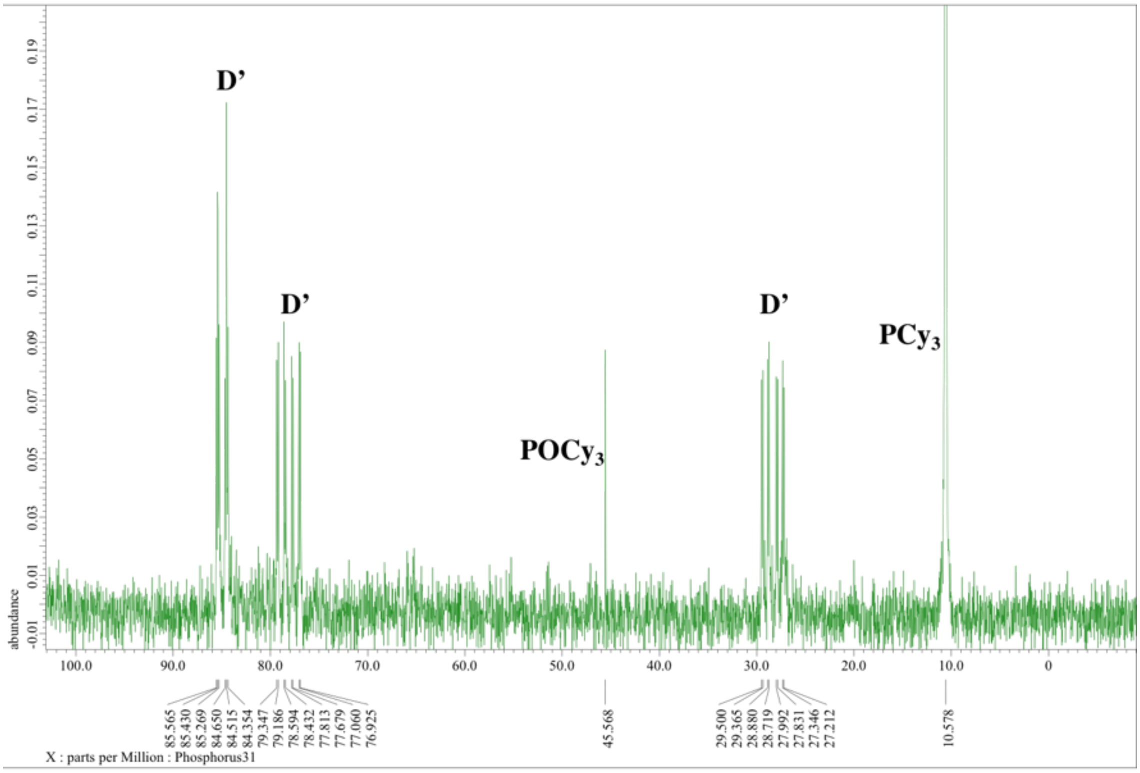

The same experiments were carried out by using RhMe(PCy_3)(dcype) **2**. Consumption of **2** was monitored by comparing the intensity of the peaks of Rh–Me moiety (δ 0.3 ppm) with that of O–Me moiety of 1,3,5-trimethoxybenzene (δ 3.3 ppm). The reaction was first order to [**2**] and inverse first order to [PCy_3]. The rate equations and the rate constant are given in eq. S4.

$$-\ln\left(\frac{[\mathbf{2}]}{[\mathbf{2}]_0}\right) = k_{\mathrm{Me1}}[\mathrm{PCy_3}]^{-1}t, \quad k_{obs} = k_{\mathrm{Me1}}[\mathrm{PCy_3}]^{-1} \qquad \text{(S4)}$$

$$k_{\mathrm{Me1}} = 1.0 \times 10^{-3}\ [\mathrm{M} \cdot \mathrm{min}^{-1}]$$

$$\text{under } \mathrm{CO_2} \text{ (1 atm at 22 ºC) at 35 ºC}$$

The detailed results were summarized in Figure S15. The representative ^{1}H NMR spectra during the measurement and the ^{31}P NMR spectrum after the measurement were provided in Figure S16 and S17.

Figure S15. Kinetic study of carboxylation of **2** in toluene-d_8.

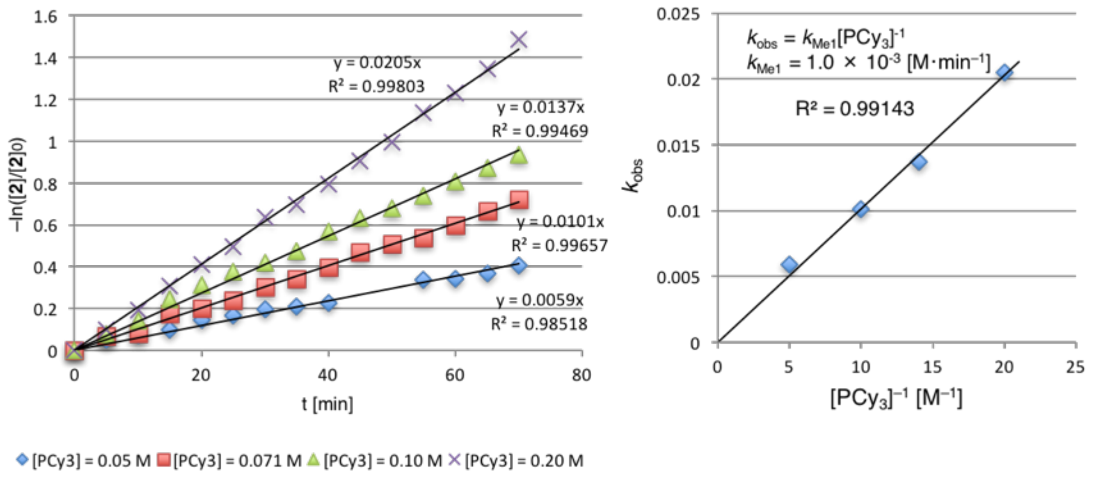

Figure S16. Kinetic study of carboxylation of **2** by ^{1}H NMR. [PCy_3] = 0.050 M. The spectra are those recorded in every 20 minutes.

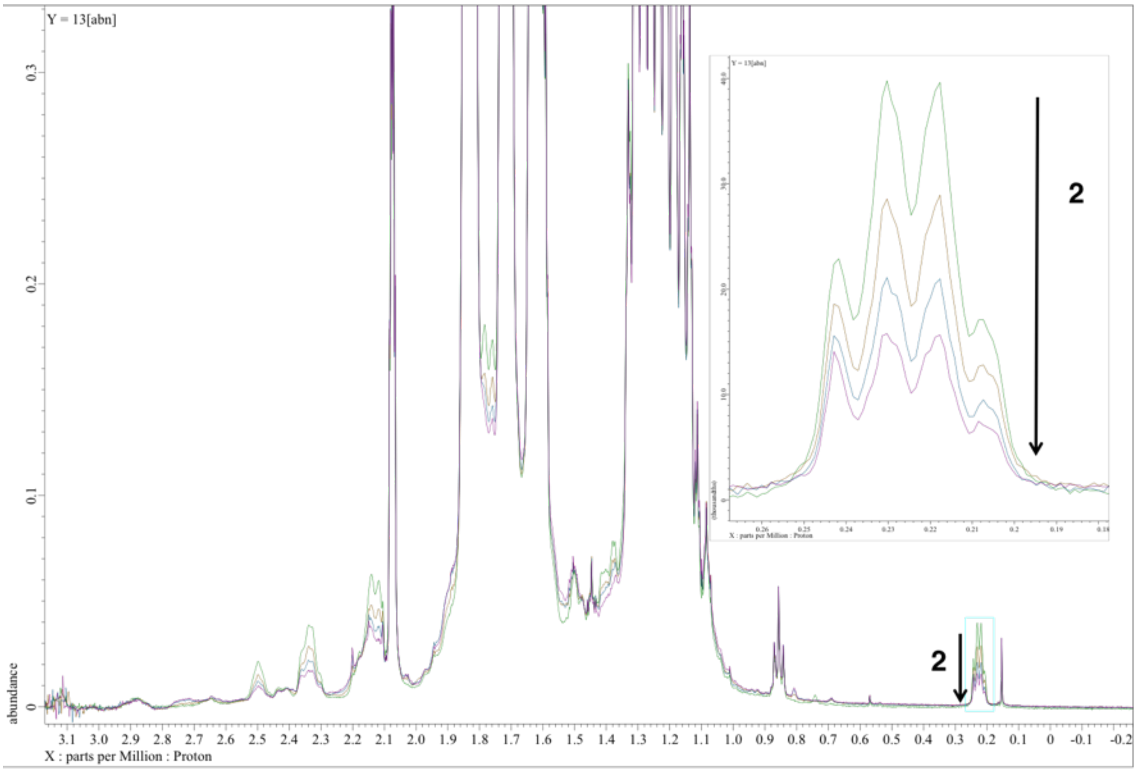

Figure S17. ^{31}P NMR spectrum after the kinetic study of carboxylation of **2** (1.25 h, [PCy_3] = 0.050 M).

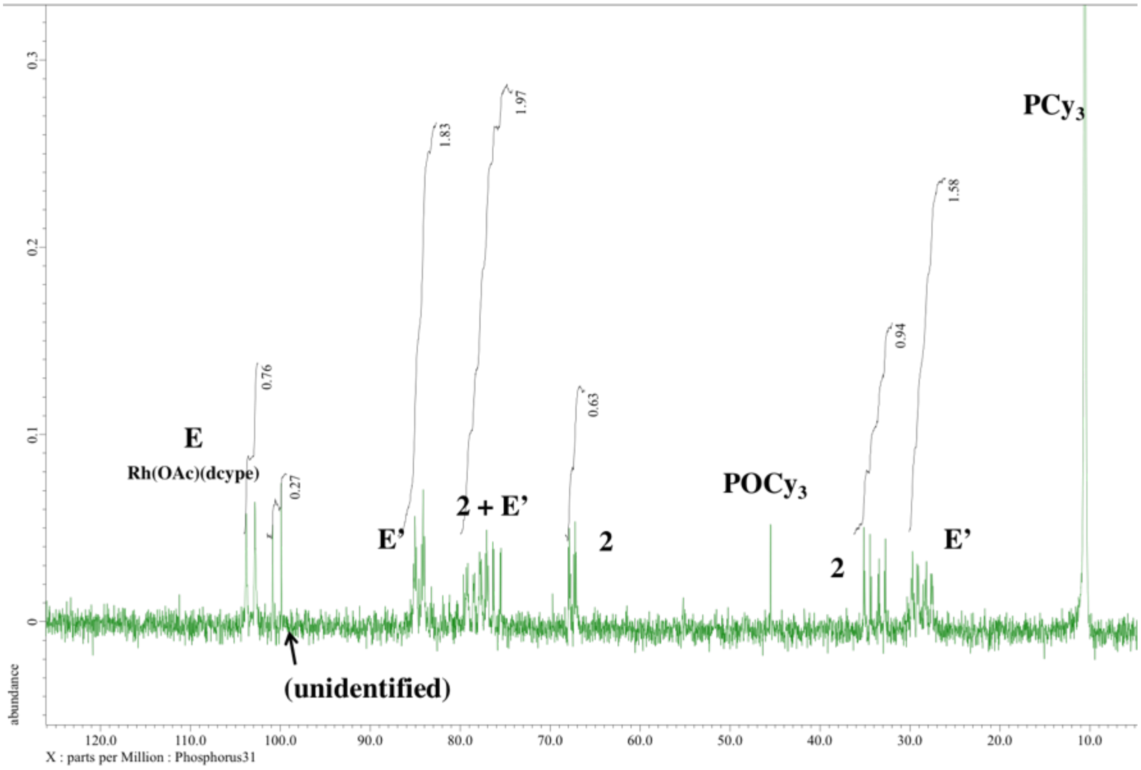

9. The Stoichiometric Reaction of 2 under CO_2 at 85 ºC (Scheme 8)

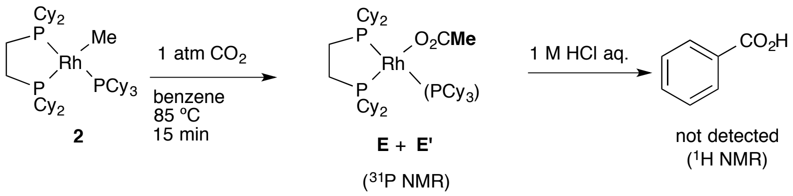

A solution of RhMe(PCy_3)(dcype) **2** (5.6 mg, 0.01 mmol) in benzene (0.5 mL) was added to an NMR tube. The system was purged with 1 atm CO_2 and then heated for 15 min at 85 ºC. After it was cooled to room temperature, the mixture was analyzed by ^{31}P NMR. Its spectrum was identical to a mixture of carboxylates Rh(O_2CR)(dcype) and Rh(O_2CR)(PCy_3)(dcype) (benzoates and acetates were difficult to be distinguished on ^{31}P NMR spectroscopy). The mixture was then treated with 1N HCl and extracted with ethyl acetate for three times. The combined organic layer was dried over $MgSO_4$, filtered and concentrated *in vacuo*. ^{1}H NMR spectrum of the mixture in $CDCl_3$ indicated no formation of benzoic acid, which supported that the complexes observed in ^{31}P NMR were both acetate Rh(OAc)(dcype) **E** and Rh(OAc)(PCy_3)(dcype) **E'**.

10. The Catalytic Carboxylation Reactions in Various Vessels (Table 1, 2)

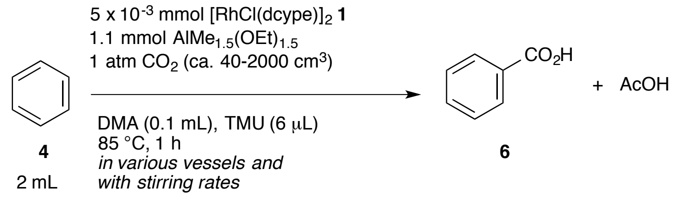

_The catalytic carboxylation reaction of benzene was carried out according to the general procedure provided in page 1 except for the reaction time of 1 h. The balloon was separately filled with CO_2 in advance and it was attached to the reaction vessel after the vessel was sufficiently purged with CO_2. After the reaction, 1N HCl (2 mL) and diethyl ether (4 mL) were added to the reaction mixture after heating under CO_2, and the mixture was stirred for 30 minutes at room temperature. Dodecane (internal standard) was added to the resulting mixture, which was diluted with methanol until it became homogeneous, and was analyzed by GC to quantify acetic acid. Diethyl ether was added to the mixture until it became heterogeneous. The organic layer was extracted with diethyl ether three times, and the extract was dried over $MgSO_4$. Removal of the solvent gave a crude mixture, of which content of benzoic acid **6** was estimated by ^{1}H NMR.

11. The *in situ*-IR Spectroscopy Experiment for The Observation of CO_2 in Toluene (Figure 5)

To a ca. 40 mL two-necked reaction vessel (test tube or round-bottom flask) equipped with an *in-situ* IR probe, degassed toluene (2 mL) was added under nitrogen atmosphere. The change in IR spectrum of the liquid phase was kept monitored during the following manipulations. After the spectrum became stabilized, the system was rapidly evacuated and purged with 1 atm CO_2 three times. The area of the peak at ca. 2350 cm^3 (an absorbance of CO_2) was recorded every 20 seconds until it reached maximum. The results are summarized in Figure S18.

Figure S18. Dissolution of CO_2 in toluene.

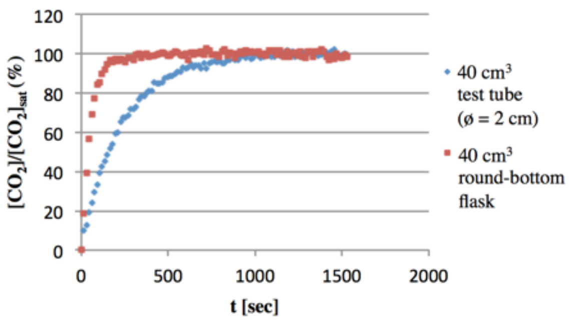

12. Analysis of A Reaction Mixture by ^{31}P NMR (Scheme 10)

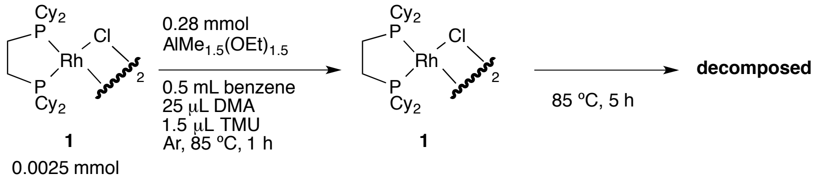

A solution of $[RhCl(dcype)]_2$ **1** (2.8 mg, 0.0025 mmol), DMA (25 μL), TMU (1.5 μL, 0.013 mmol) and $AlMe_{1.5}(OEt)_{1.5}$ (33 mg, 0.28 mmol) in benzene (0.5 mL) was added to an NMR tube with a sealed glass tube which contained a benzene solution of PPh_3 as an internal standard. The mixture was analyzed by ^{31}P NMR to estimate the initial content of **1** by comparing intensities of the peaks of **1** and PPh_3. The mixture was heated at 85 ºC in an oil bath, and its ^{31}P NMR spectra were recorded after 1 h and 6 h. No significant change was observed after 1 h, but ca. 70 % of **1** was consumed to give many unidentified species after 6 h. Alternatively, almost no change was also observed after heating for 1 h when the reaction was carried out under CO_2.

13. The Reaction of $Rh(O_2CPh)(dcype)$ D with $AlMe_{1.5}(OEt)_{1.5}$ (Scheme 11)

In a glovebox, $Rh(O_2CPh)(dcype)$ **D** (2.9 mg, 5×10^{-3} mmol) was dissolved in benzene (0.5 mL). A solution of $AlMe_{1.5}(OEt)_{1.5}$ (13 mg, 0.1 mmol) in benzene (0.2 mL) was added to this solution. The color of the mixture changed from yellow to orange, and then to dark yellow. ^{31}P NMR analysis of the mixture suggested decomposition of **D** to give small amounts of unidentified complexes.

14. The Reaction of $Rh(O_2CPh)(dcype)$ D with $AlMe_{1.5}(OEt)_{1.5}$ in The Presence of $AlClMe_2$ (Scheme 11)

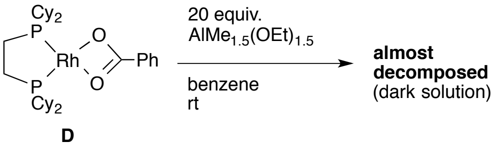

In a glovebox, a solution of $Rh(O_2CPh)(dcype)$ **D** (2.9 mg, 5×10^{-3} mmol) in benzene (0.5 mL) was added to an NMR tube with a sealed glass tube which contained a benzene solution of PPh_3 as an internal standard. The mixture was analyzed by ^{31}P NMR to estimate the initial content of **D**, by comparing intensities of the peaks of **D** and PPh_3. After this, a mixture of $AlMe_{1.5}(OEt)_{1.5}$ (13 mg, 0.1 mmol) and $AlClMe_2$ (1.0 M hexane solution, 5 μL, 5×10^{-3} mmol) in benzene (0.2 mL) was added. A color of the mixture slightly changed from yellow to orange after the addition, but it turned again to yellow soon. ^{31}P NMR analysis of the mixture suggested complete conversion of **D** to

[RhCl(dcype)]$_2$ **1**. The yield was estimated to 60–70 % according to relative intensity of the peaks of **1** to those of PPh_3.

15. The catalytic carboxylation of benzene using Rh(O$_2$CPh)(dcype) D

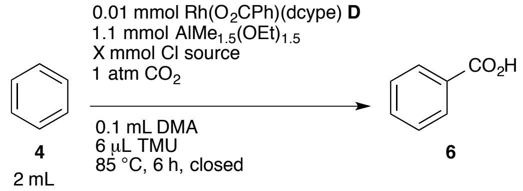

The reaction was carried out according to the general procedure provided in page 1 except Rh(O$_2$CPh)(dcype) **D** was used instead of [RhCl(dcype)]$_2$ **1** and the chloride source ($AlMe_2Cl$ or NBu_4Cl, 0.01-0.05 mmol) was added simultaneously with $AlMe_{1.5}(OEt)_{1.5}$. The results are summarized in Table S1.

Table S1. Effect of chloride source in the carboxylation reaction by Rh(O$_2$CPh)(dcype) **D.**

entry	additive	X	TON
1	none	-	2.5
2	$AlMe_2Cl$	0.01	27
3	$AlMe_2Cl$	0.05	2.6
4	Bu_4NCl	0.01	7.7

16. Methylation of RhCl(PCy$_3$)(dcype) F with AlMe$_3$ (ref. 24)

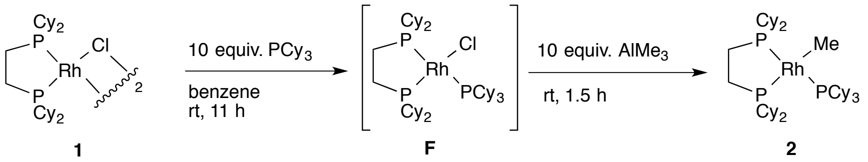

In a glovebox, [RhCl(dcype)]$_2$ **1** (2.8 mg, 2.5 x 10^{-3} mmol) and PCy_3 (14 mg, 0.05 mmol) were dissolved in benzene (0.5 mL) and the mixture was stood at room temperature. After 11 h, the reaction mixture was analyzed by ^{31}P NMR. The peaks of **1** almost disappeared and new three ddd peaks were observed at δ 83, 80, 27 ppm, suggesting the formation of RhCl(PCy$_3$)(dcype) **F** (detailed values are shown below), which had nonequivalent three phosphines. Then, the mixture was treated with $AlMe_3$ (neat, 4.8 μL, 0.05 mmol) in a glovebox at room temperature. After 1.5 h, the mixture was analyzed again by ^{31}P NMR. The peaks of **F** completely disappeared and **2** was observed as a major product. Its ^{31}P NMR spectrum was consistent with those of the authentic compound prepared as described above.

^{31}P NMR spectrum of RhCl(PCy$_3$)(dcype) **F** in benzene: δ 83.3 (ddd, $J_{P\text{-}Rh}$ = 188 Hz, $J_{P\text{-}P}$ = 31, 30 Hz), 80.3 (ddd, $J_{P\text{-}Rh}$ = 140 Hz, $J_{P\text{-}P}$ = 323, 31 Hz), 27.4 (ddd, $J_{P\text{-}Rh}$ = 118 Hz, $J_{P\text{-}P}$ = 323, 30 Hz).

17. References

[i]: (a) Van der Ent, A.; Onderdelingen, A. L.; Schunn, R. A. *Inorg. Synth.*, **1973**, *14*, 92–95. (b) Giordano, G.; Crabtree, R. H. *Inorg. Synth.* **1979**, *19*, 218–220. (c) Chatt, J.; and Venanzi, L. M. *J. Chem. Soc.*, **1957**, 4735.

[ii]: Suga, T.; Mizuno, H.; Takaya, J.; Iwasawa, N. *Chem. Commun.*, **2014**, *50*, 14360–14363.

[iii]: We have not determined whether rhodium carboxylates **D** and **E** are monomeric or dimeric completely. However, their FAB$^+$ mass spectra indicate they are monomeric. In addition, related Rh(OAc)(P^{i}Pr$_3$)$_2$ has monomeric structure in the solid state. See: Werner, H.; Martin, S.; Oliver, N.; Wolf, J. *Chem. Ber.* **1994**, *127*, 27–38.

[iv]: Ochiai, H.; Jang, M.; Hirano, K.; Yorimitsu, Y.; Oshima, K. *Org. Lett.* **2008**, *10*, 2681–2683.

^{1}H NMR spectra of RhMe(PCy$_3$)(dcype) **2**.

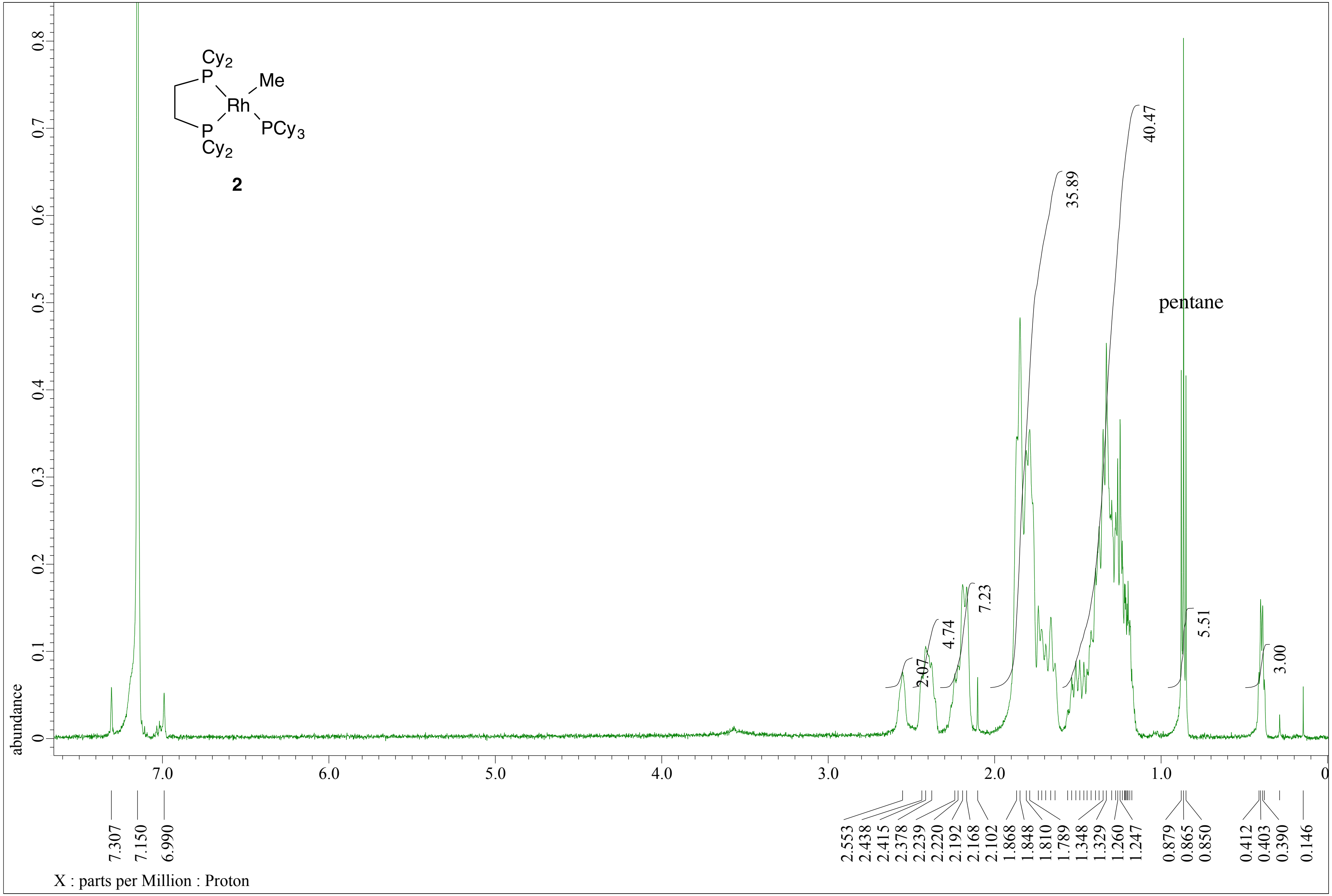

^{13}C NMR spectra of $RhMe(PCy_3)(dcype)$ **2**. Minor peaks correspond to $RhPh(PCy_3)(dcype)$ **3**, generated during measurement.

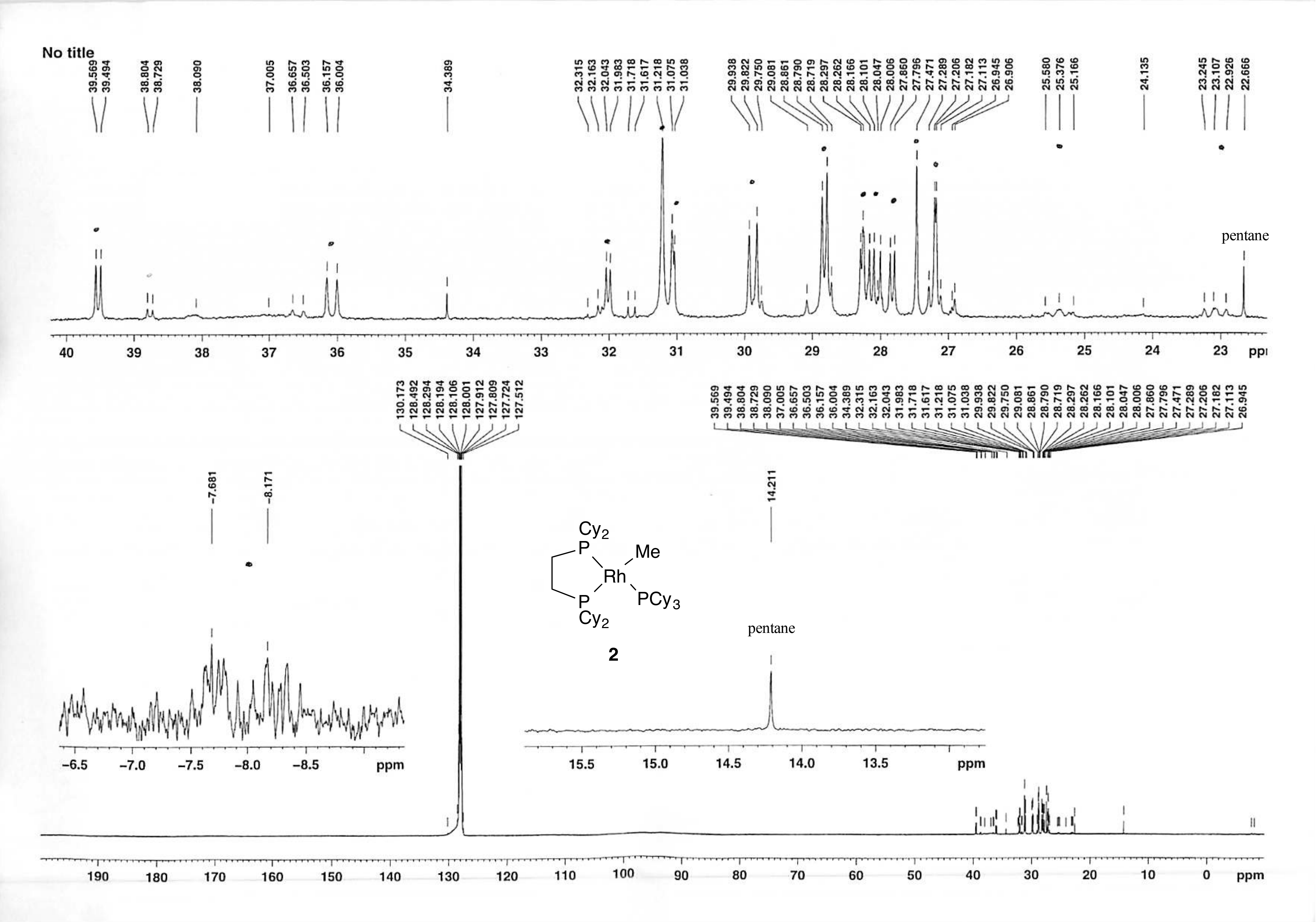

^{31}P NMR spectra of $RhMe(PCy_3)(dcype)$ **2**.

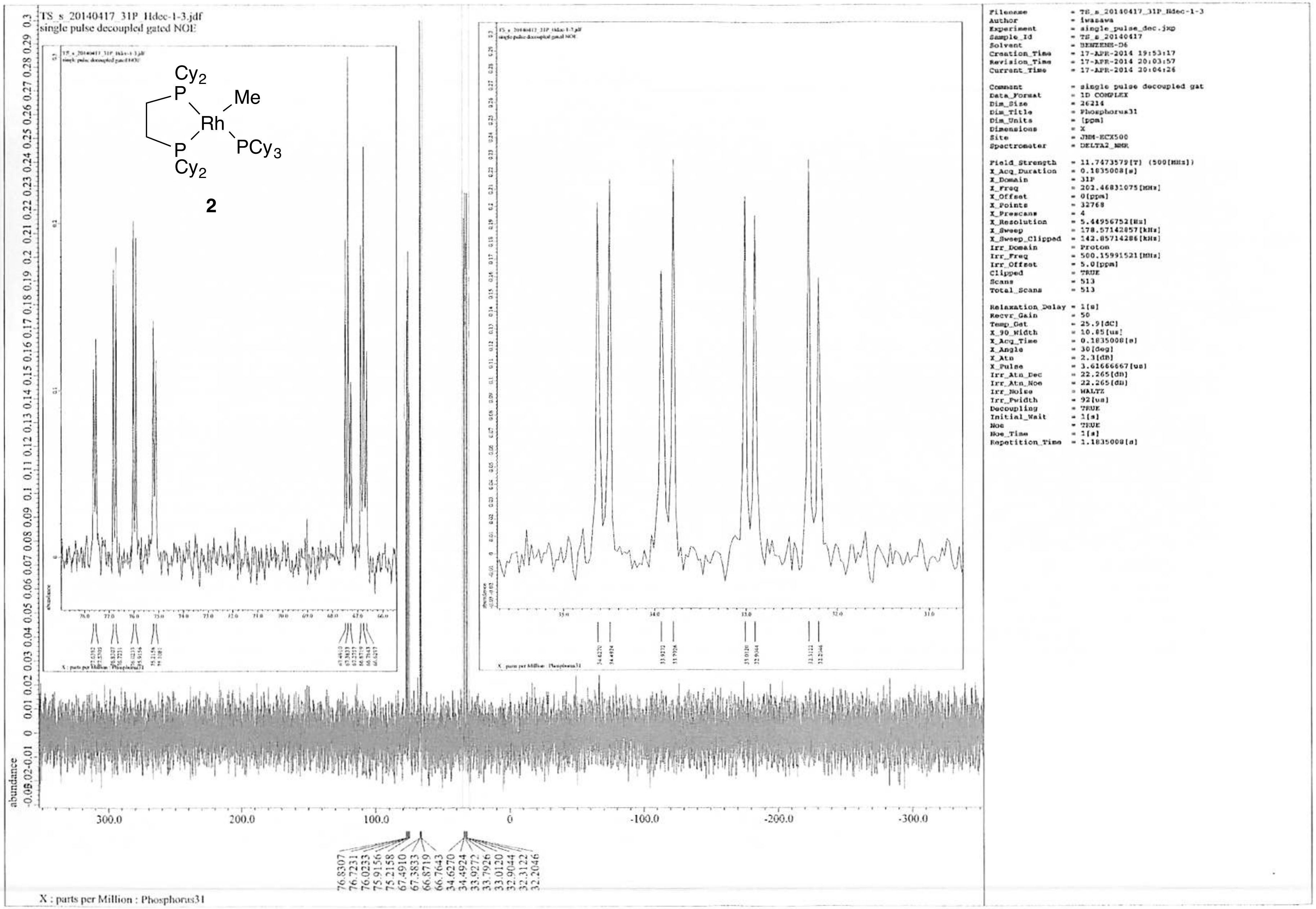

^{1}H NMR spectra of RhPh(PCy$_3$)(dcype) **3**.

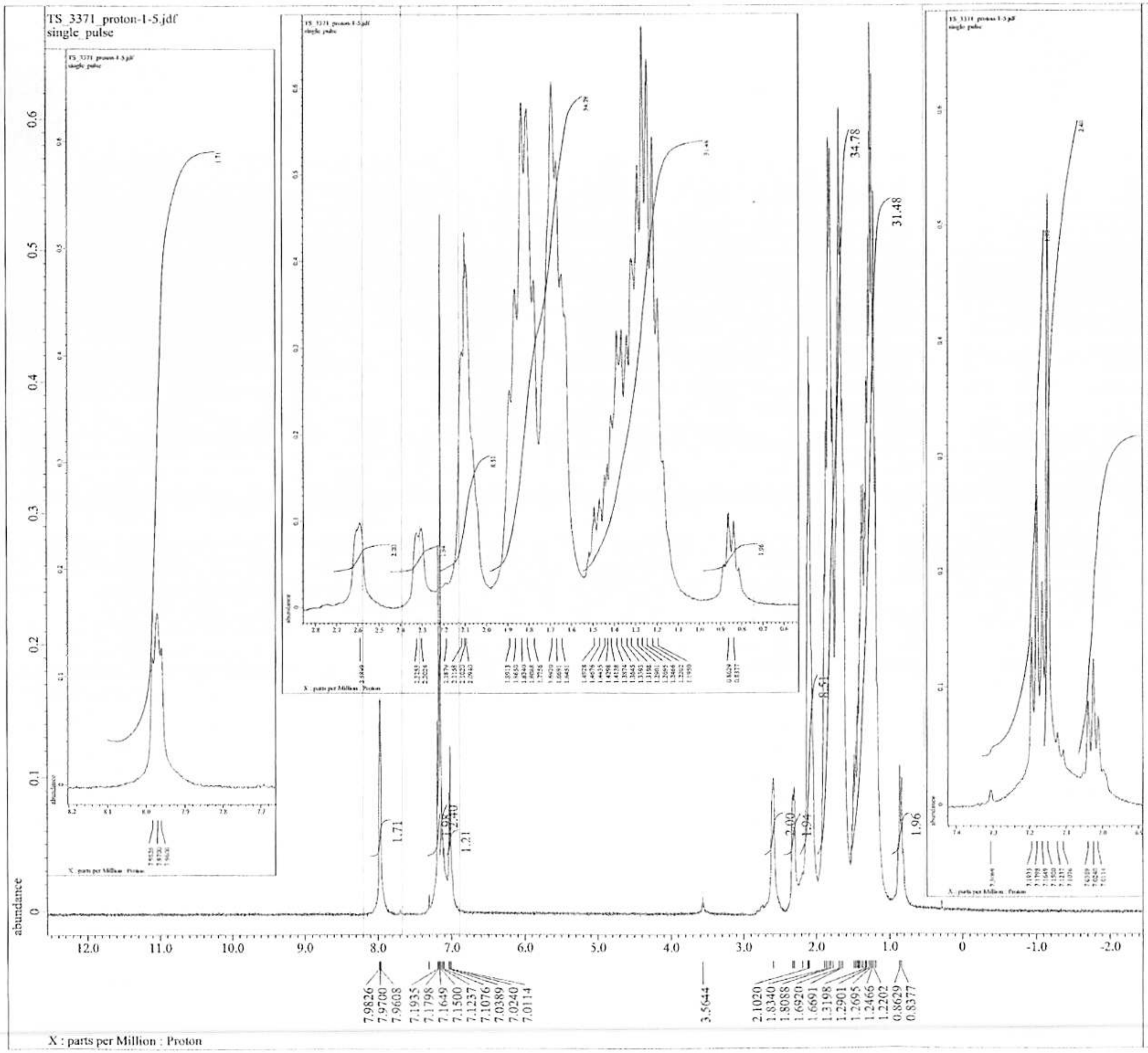

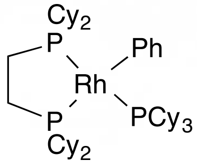

3

^{13}C NMR spectra of RhPh(PCy_3)(dcype) **3**.

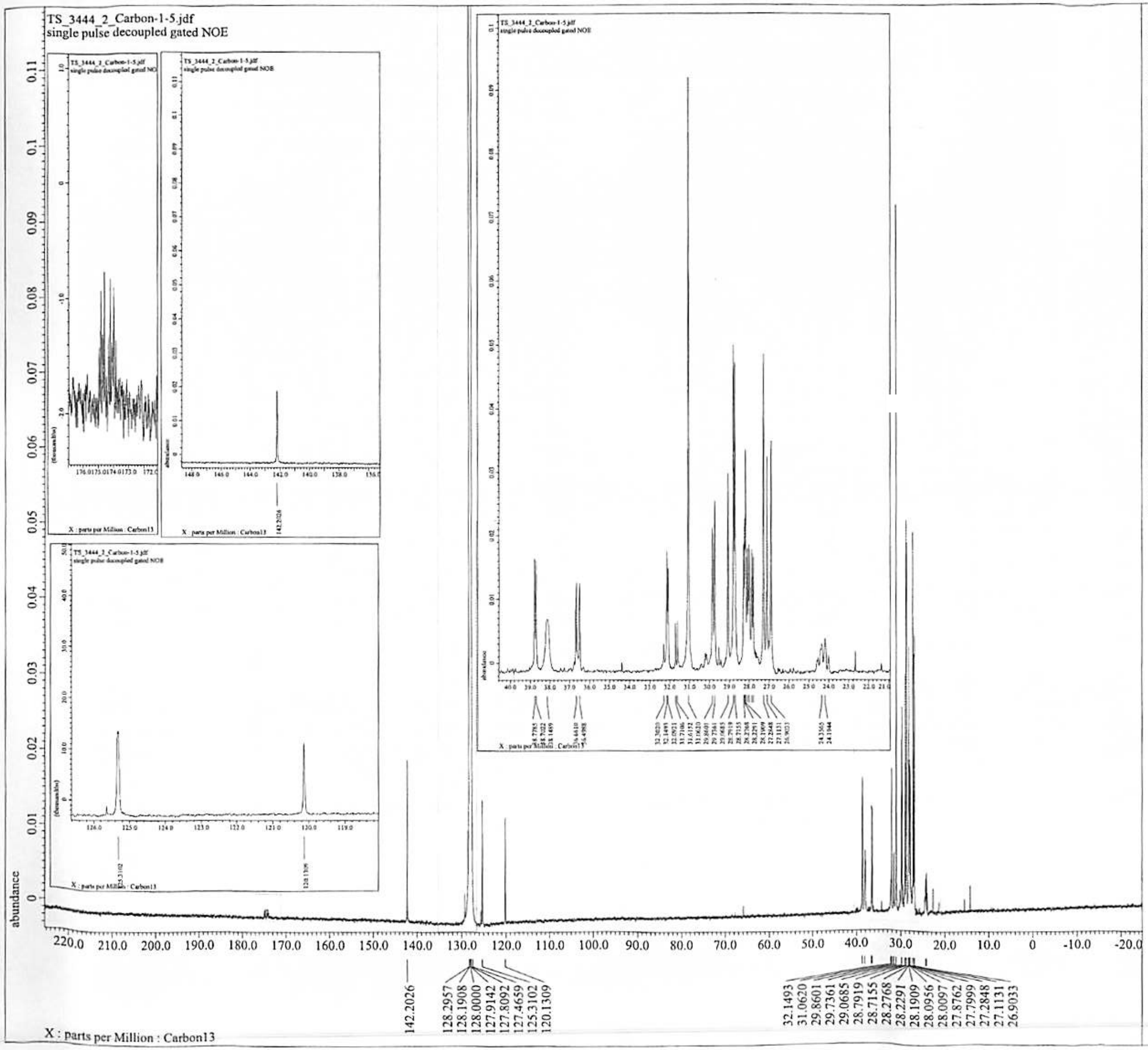

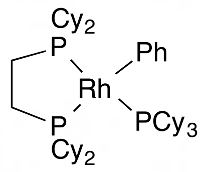

3

^{31}P NMR spectra of $RhPh(PCy_3)(dcype)$ **3**.

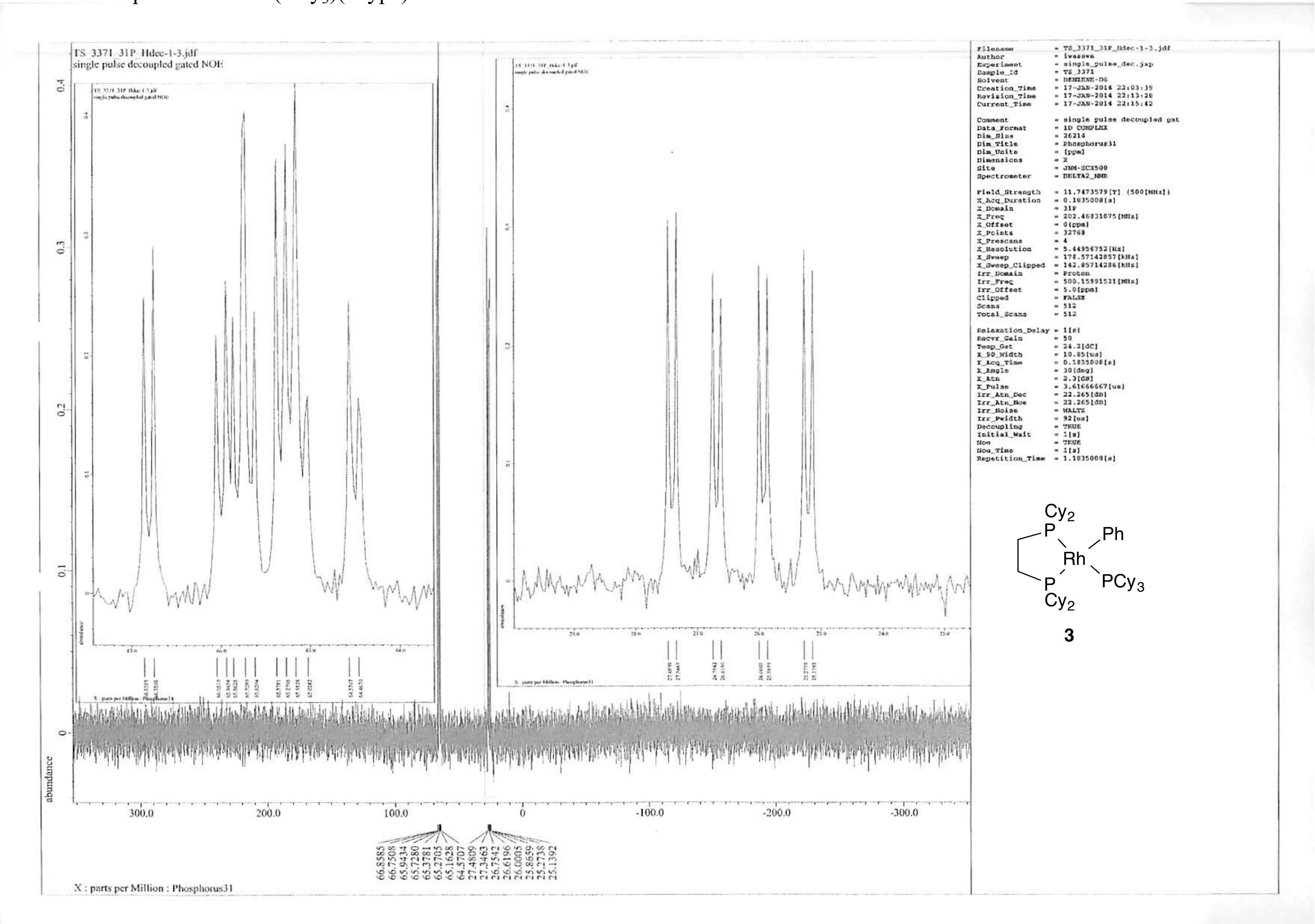

^{1}H NMR spectra of $[Rh(O_2CPh)(cod)]_2$

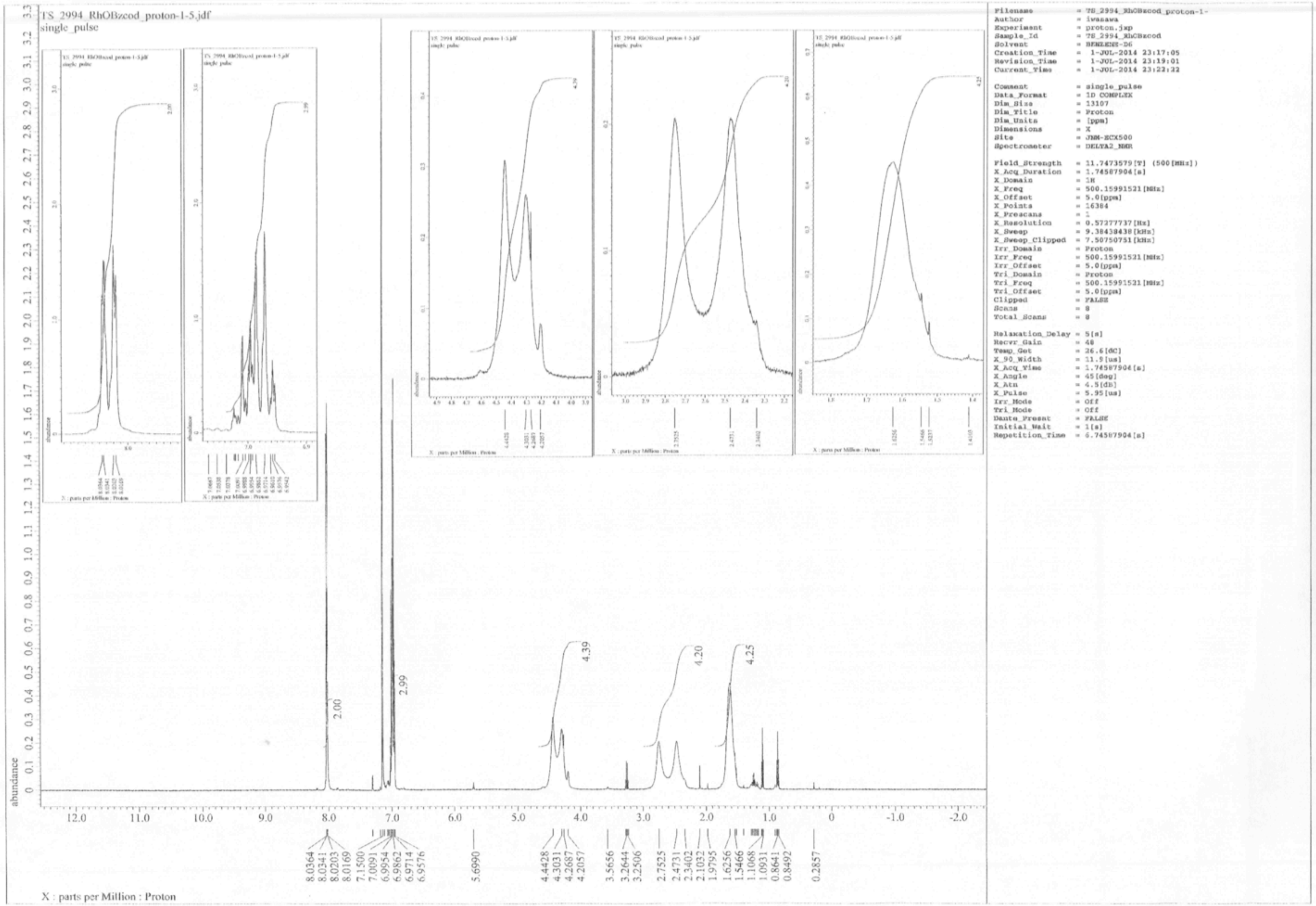

^{1}H NMR spectra of $Rh(O_2CPh)(dcype)$ **D**.

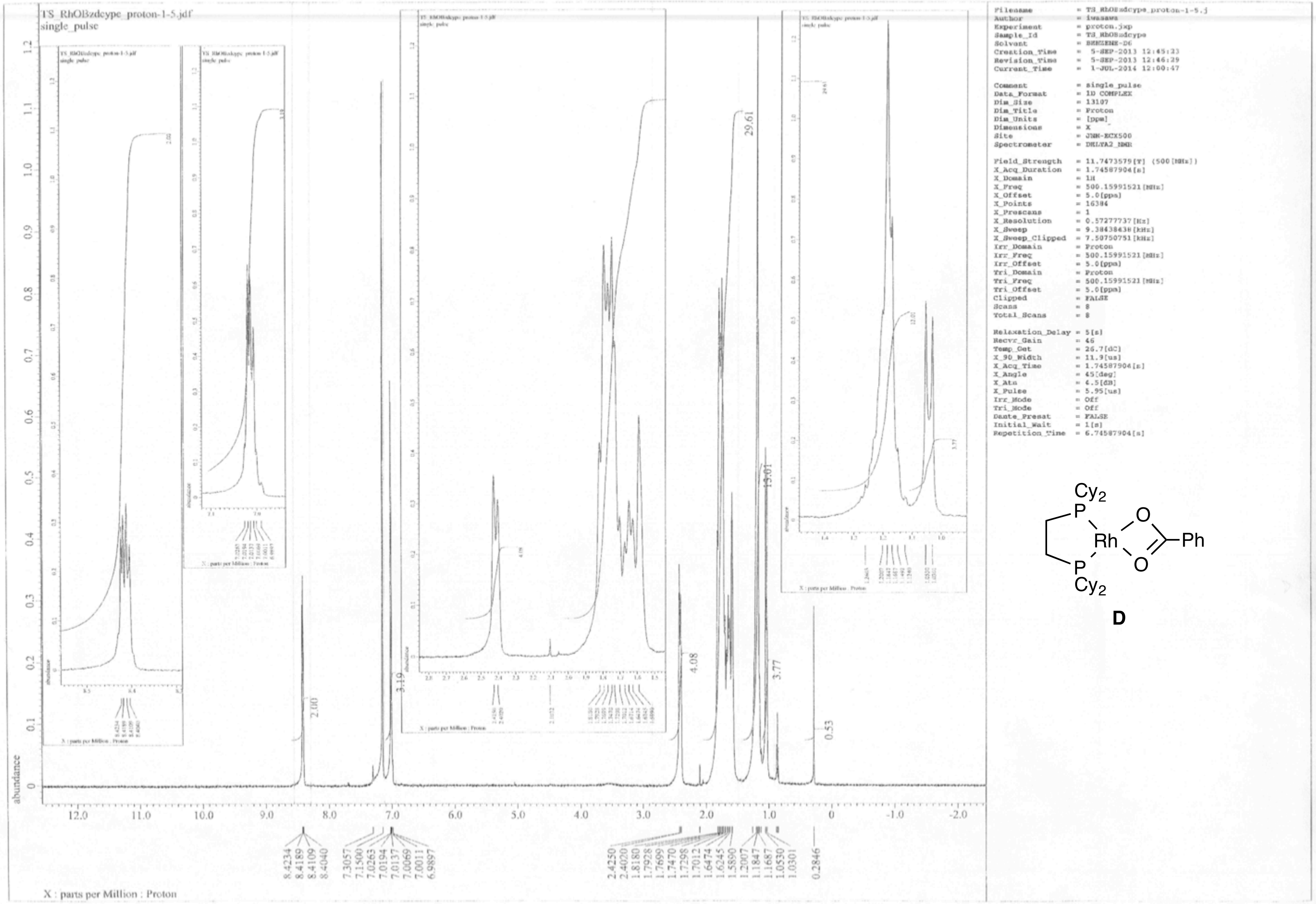

^{13}C NMR spectra of Rh(O_2CPh)(dcype) **D**.

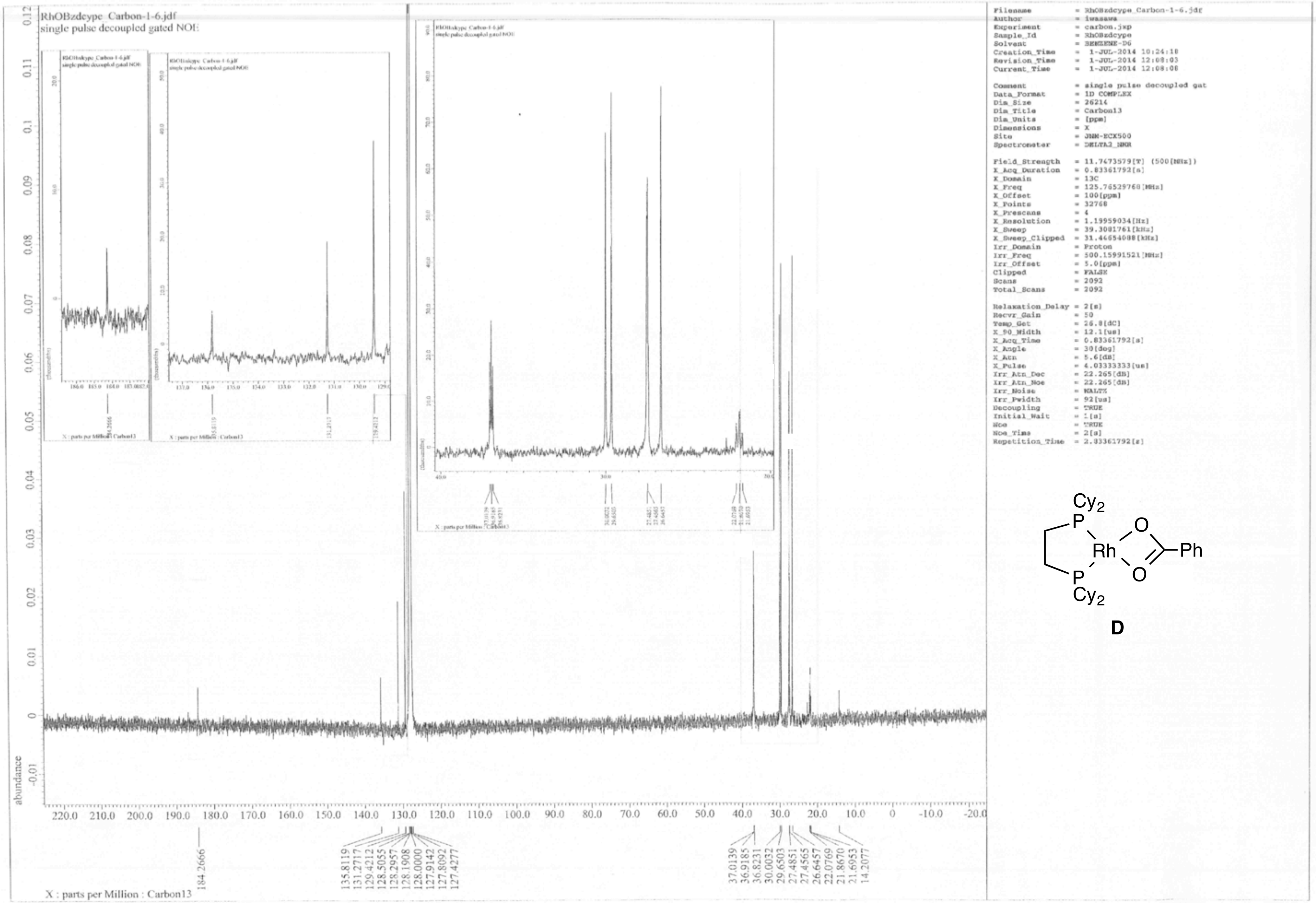

D

^{31}P NMR spectra of Rh(O$_2$CPh)(dcype) **D**.

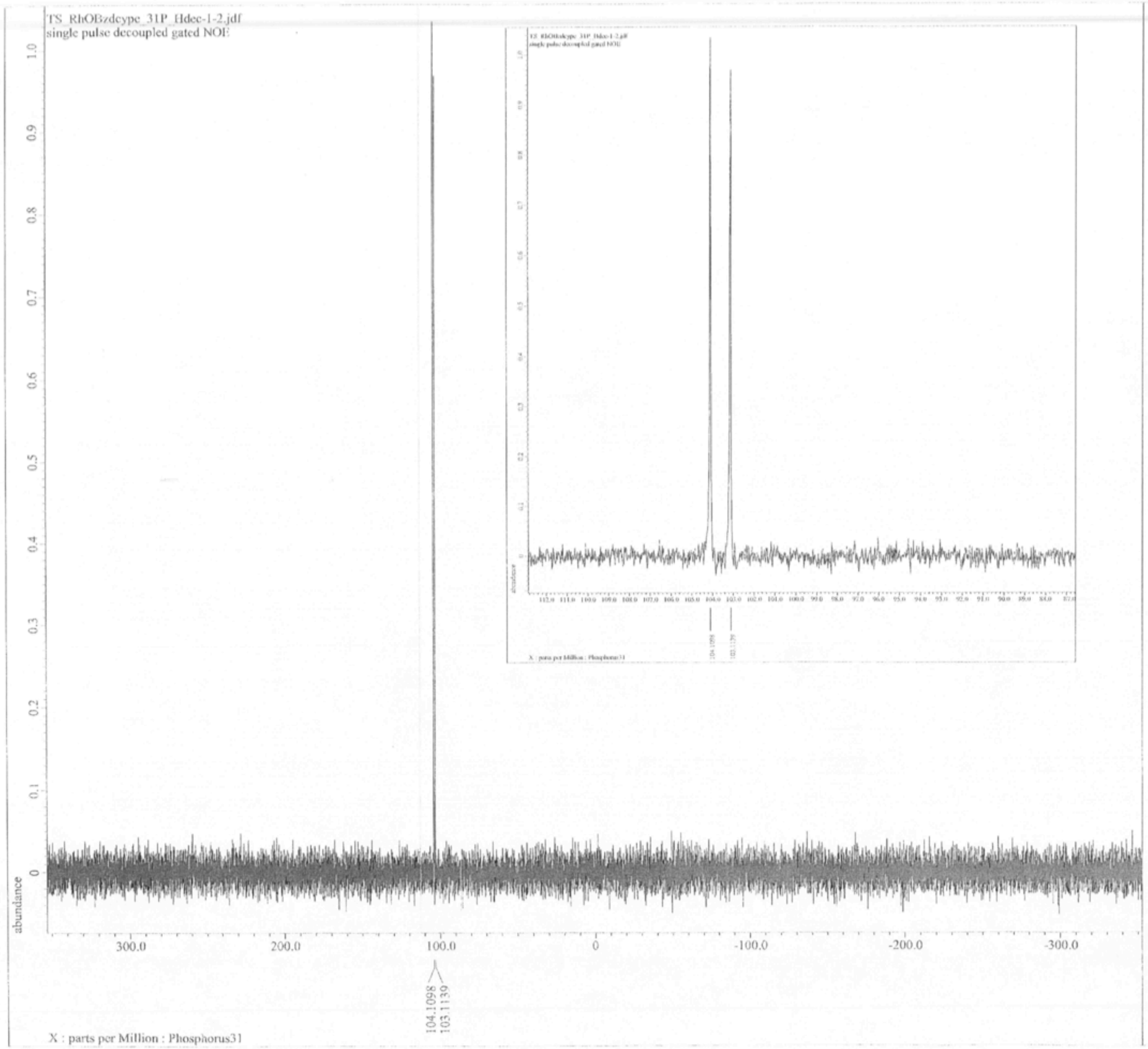


```
Filename            = TS_RhOBzdcype_31P_Hdec-1-2
Author              = iwasawa
Experiment          = single_pulse_dec.jxp
Sample_Id           = TS_RhOBzdcype
Solvent             = BENZENE-D6
Creation_Time       =  5-SEP-2013 12:46:40
Revision_Time       =  1-JUL-2014 11:58:42
Current_Time        =  1-JUL-2014 12:00:59

Comment             = single pulse decoupled gat
Data_Format         = 1D COMPLEX
Dim_Size            = 26214
Dim_Title           = Phosphorus31
Dim_Units           = [ppm]
Dimensions          = X
Site                = JNM-ECX500
Spectrometer        = DELTA2_NMR

Field_Strength      = 11.7473579[T] (500[MHz])
X_Acq_Duration      = 0.1835008[s]
X_Domain            = 31P
X_Freq              = 202.46831075[MHz]
X_Offset            = 0[ppm]
X_Points            = 32768
X_Prescans          = 4
X_Resolution        = 5.44956752[Hz]
X_Sweep             = 178.57142857[kHz]
X_Sweep_Clipped     = 142.85714286[kHz]
Irr_Domain          = Proton
Irr_Freq            = 500.15991521[MHz]
Irr_Offset          = 5.0[ppm]
Clipped             = FALSE
Scans               = 132
Total_Scans         = 132

Relaxation_Delay    = 1[s]
Recvr_Gain          = 50
Temp_Get            = 27.1[dC]
X_90_Width          = 10.85[us]
X_Acq_Time          = 0.1835008[s]
X_Angle             = 30[deg]
X_Atn               = 2.3[dB]
X_Pulse             = 3.61666667[us]
Irr_Atn_Dec         = 22.265[dB]
Irr_Atn_Noe         = 22.265[dB]
Irr_Noise           = WALTZ
Irr_Pwidth          = 92[us]
Decoupling          = TRUE
Initial_Wait        = 1[s]
Noe                 = TRUE
Noe_Time            = 1[s]
Repetition_Time     = 1.1835008[s]
```

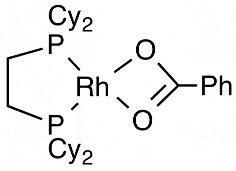

D

^{1}H NMR spectra of Rh(OAc)(dcype) **E**.

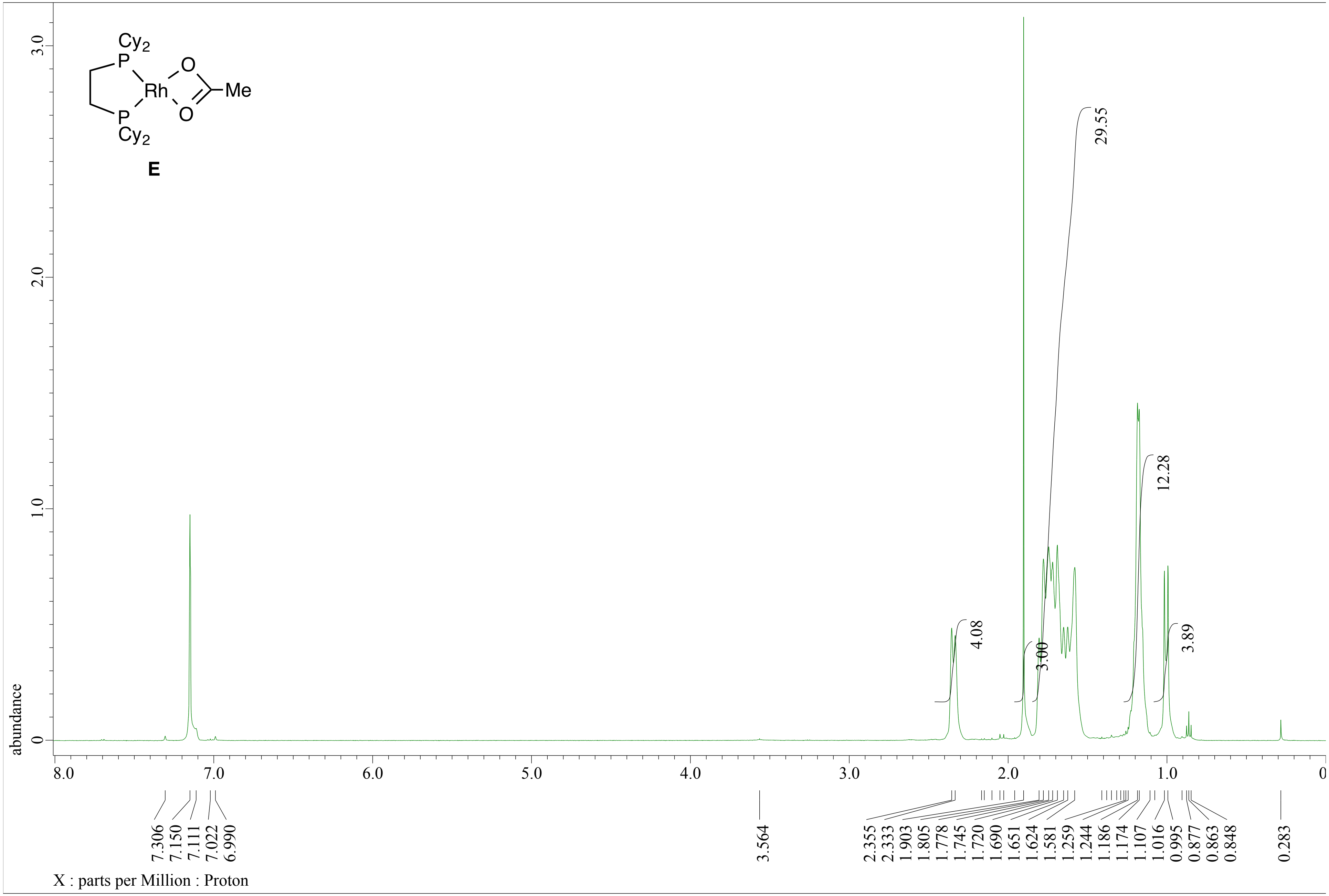

^{13}C NMR spectra of Rh(OAc)(dcype) **E**.

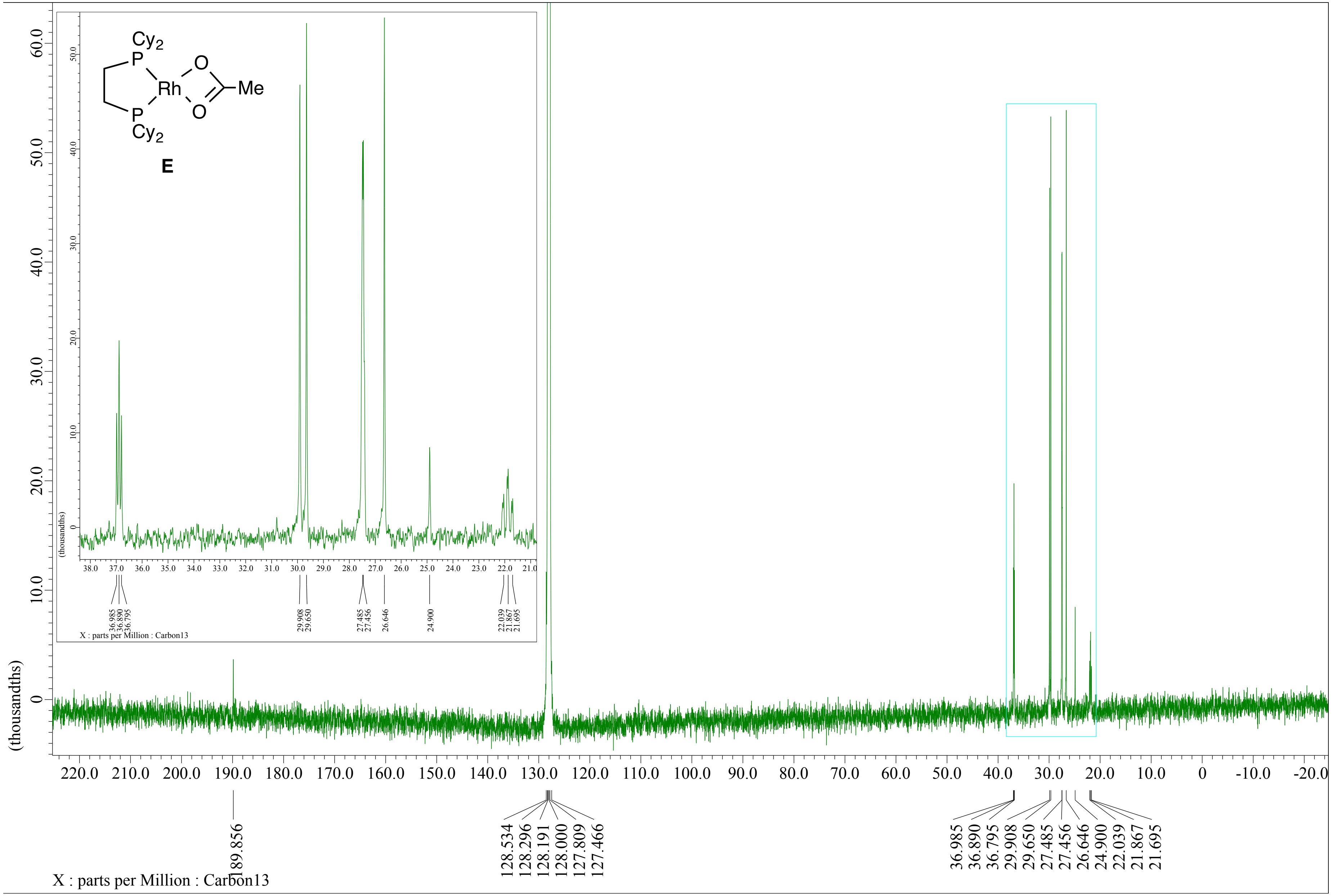

^{31}P NMR spectra of Rh(OAc)(dcype) **E**.

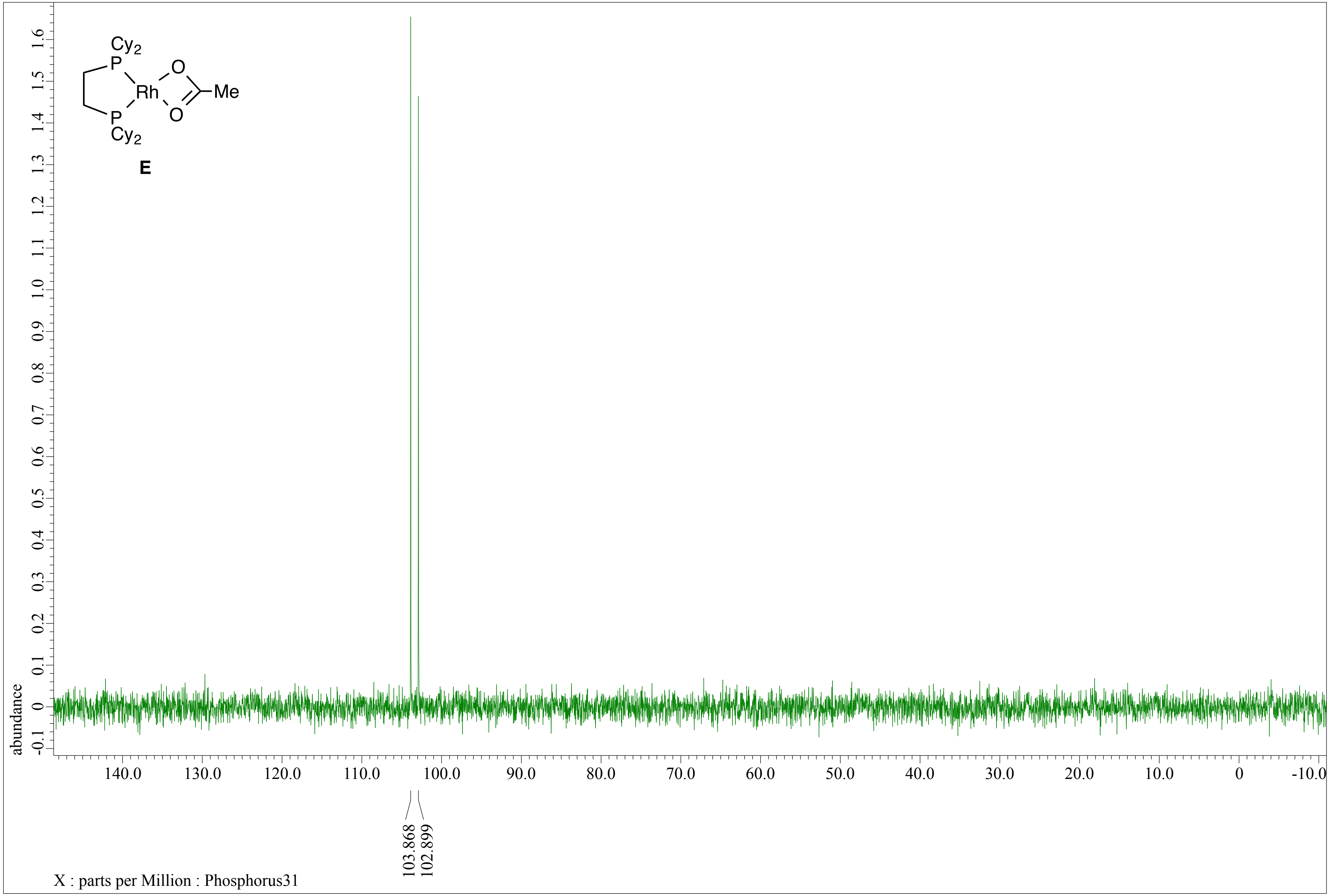

Supplement: Supplementary file 1 [file SC-008-C6SC03838G-s001.pdf]
